# Positive feedback in Ras activation by full-length SOS arises from autoinhibition release mechanism

**DOI:** 10.1016/j.bpj.2024.07.014

**Published:** 2024-07-16

**Authors:** He Ren, Albert A. Lee, L.J. Nugent Lew, Joseph B. DeGrandchamp, Jay T. Groves

**Affiliations:** 1 Department of Chemistry, University of California Berkeley, Berkeley, California; 2 Department of Molecular and Cell Biology, University of California Berkeley, Berkeley, California; 3 Division of Molecular Biophysics and Integrated Bioimaging, Lawrence Berkeley National Laboratory, Berkeley, California

## Abstract

Signaling through the Ras-MAPK pathway can exhibit switch-like activation, which has been attributed to the underlying positive feedback and bimodality in the activation of RasGDP to RasGTP by SOS. SOS contains both catalytic and allosteric Ras binding sites, and a common assumption is that allosteric activation selectively by RasGTP provides the mechanism of positive feedback. However, recent single-molecule studies have revealed that SOS catalytic rates are independent of the nucleotide state of Ras in the allosteric binding site, raising doubt about this as a positive feedback mechanism. Here, we perform detailed kinetic analyses of receptor-mediated recruitment of full-length SOS to the membrane while simultaneously monitoring its catalytic activation of Ras. These results, along with kinetic modeling, expose the autoinhibition release step in SOS, rather than either recruitment or allosteric activation, as the underlying mechanism giving rise to positive feedback in Ras activation.

## Significance

Positive feedback is a prominent feature in the activation of Ras by SOS, and Ras binding to an allosteric site on SOS has long been considered a mechanism of this positive feedback. However, more recent experimental investigations of SOS allosteric activation have failed to reveal such effects. Detailed kinetic analyses of the full-length SOS protein now reveal its unique autoinhibition release process to be the primary source of positive feedback.

## Introduction

Ras is a membrane-bound small GTPase that cycles between a GTP-bound on-state and a GDP-bound off-state (1). In the GTP-bound state, Ras can recruit downstream effector proteins to the membrane, leading to activation of the MAPK and PI3K pathways, which ultimately leads to cell growth and proliferation (2). Under nonactivating conditions, Ras resides in the GDP-bound state. Ras activation to the GTP-bound state is controlled by guanine nucleotide exchange factors (GEFs) that catalyze exchange of Ras-bound nucleotide with free nucleotide from the cytosol, which is primarily GTP (3). In the cellular setting, bimodality, or switch-like activity, in the Ras-MAPK signaling pathway has been shown to be important in lymphocytes (4) and contributes to the sharp boundary that discriminates positive and negative selection of thymocytes (5). Ras misregulation is a common cause of cancers, including lung cancer, colorectal cancer, and pancreatic cancer (6,7,8).

**Figure 1:**
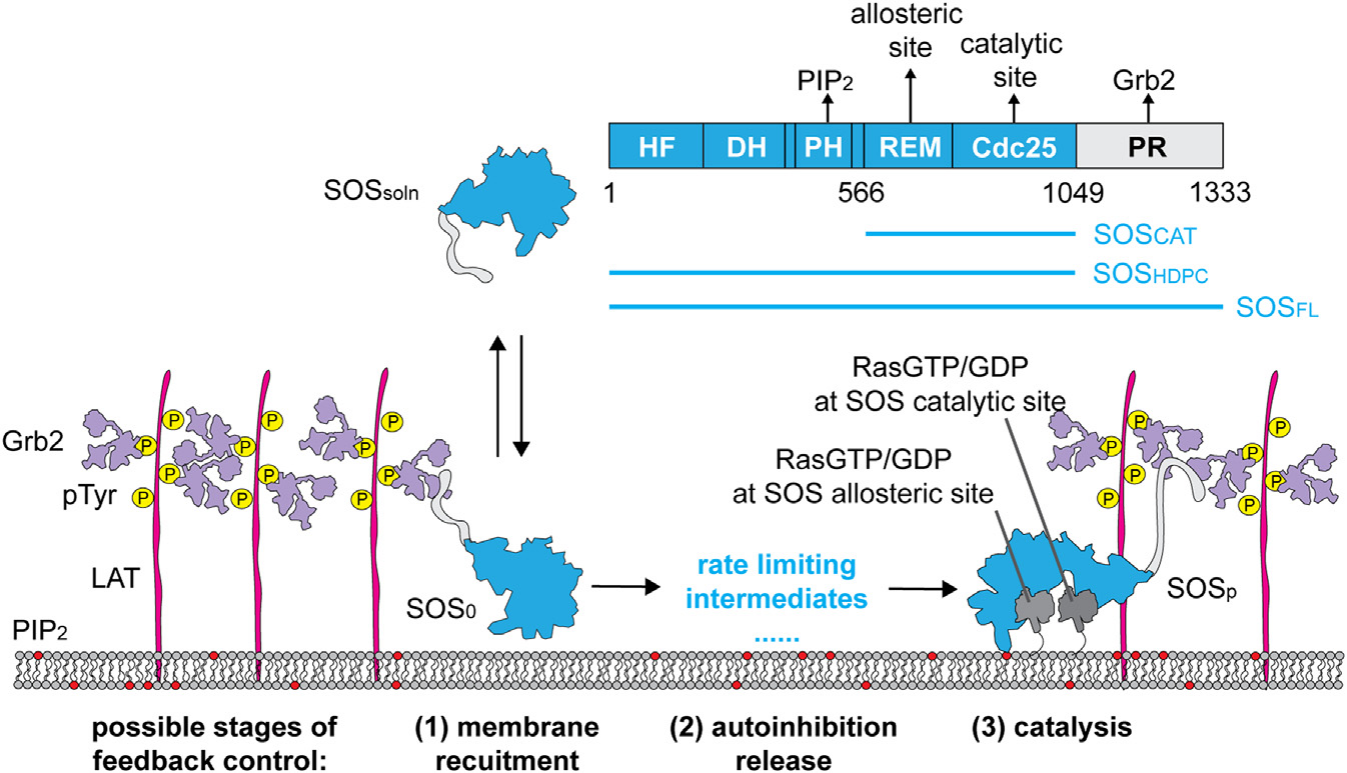
The domain organization and activation process of SOS. SOS_FL_ stays in an autoinhibited conformation in solution (SOS_soln_). After T cell receptor stimulation and LAT phosphorylation, SOS_FL_ activation follows initial membrane recruitment in an autoinhibited conformation (SOS_0_), slow autoinhibition release on the membrane, and processive Ras nucleotide exchange after activation (SOS_p_). These three steps of activation provide potential mechanisms for feedback control in SOS.

Son of Sevenless (SOS) is a key RasGEF, which operates downstream of both the growth factor receptor and T cell receptor pathways. The amine terminus of SOS consists of three regulatory domains: a histone fold (HF) domain (9), a Dbl homology (DH) domain, and a Pleckstrin homology (PH) domain (10). The REM-Cdc25 catalytic core sits in the middle (11) and is followed by a carboxy-terminal, structurally disordered proline-rich (PR) tail (12). Upon receptor activation, SOS is recruited via Grb2 to the cytosolic tail of epidermal growth factor receptor (EGFR) or, in the case of T cell receptor signaling, to Linker for activation of T cells (LAT). In both cases, SH3 domains on the adaptor protein Grb2 bind to the PR domain of SOS, while the Grb2 SH2 domain binds to phospho-tyrosine sites on the active receptor or scaffold (13,14). Once on the membrane, SOS can initiate the nucleotide exchange reaction after a multistep autoinhibition release process (Fig. 1) (15).

An unusual feature of SOS, compared with other RasGEFs, is that it has two Ras binding sites in its REM-Cdc25 catalytic core (16). There is one catalytic site at the Cdc25 domain and one noncatalytic allosteric site positioned between the REM and Cdc25 domains (11,15). Structural studies have shown that allosteric Ras binding is essential for SOS catalytic activity (17,18). In addition, both solution and liposome nucleotide exchange assays using the catalytic core of SOS have shown positive feedback in the activation of Ras by SOS. The higher binding affinity for RasGTP relative to RasGDP at the SOS allosteric site as well as a presumption of nucleotide-specific enhancement of catalytic activity have generally been accepted as the mechanisms of positive feedback (4,18,19,20,21). However, these observations were made with truncated SOS constructs lacking autoinhibition and membrane recruitment through accessory domains. Recent studies have started to provide detailed characterizations of the full-length SOS (SOS_FL_) molecule (22,23).

Single-molecule membrane microarray studies of SOS have revealed several facets of its functional mechanism that were not evident in earlier work. First, SOS is highly processive and can activate hundreds of Ras molecules in a single membrane binding event (22,24,25). Second, the catalytic rate of Ras activation by an individual SOS molecule is independent of the nucleotide state of Ras in the allosteric binding site (24). Third, SOS_FL_ exhibits an autoinhibition release process on the membrane that introduces a long delay (tens of seconds) between initial recruitment of SOS to the membrane and onset of its processive catalytic activity. Analyses of the delay time indicates that this slow activation process consists of multiple steps occurring far from equilibrium (23). This slow autoinhibition release feature of SOS enables a novel type of kinetic proofreading regulation of SOS activity, which is under the control of a protein condensation phase transition of the recruiting EGFR or LAT molecules (23,26,27). Collectively, this body of work experimentally demonstrates that SOS_FL_ lacks the classically presumed positive feedback in allosteric enhancement of its catalytic rate. SOS_FL_ does, however, exhibit marked positive feedback in Ras activation (28), raising questions of what the actual underlying mechanism is.

In this study, we performed detailed kinetic analyses of receptor-mediated recruitment and activity of SOS_FL_ in a supported membrane platform. Using total internal reflection fluorescence (TIRF) microscopy, we monitor the recruitment (via Grb2) of SOS_FL_ to phosphorylated LAT scaffold molecules on the membrane while simultaneously tracking the Ras activity state using a modified Ras binding domain from Raf-1 (residues 56–131, K65E; referred to as RBD hereafter). This RBD construct exhibits substantially faster binding kinetics compared with wild-type and provides faithful real-time readout of RasGTP densities on the membrane (23). The results confirm that receptor-mediated recruitment of SOS_FL_ is independent of the Ras-nucleotide state. Furthermore, we obtain quantitative measurements of the effective catalytic rate of a defined ensemble of membrane-recruited SOS_FL_ molecules as a function of RasGTP density, which confirms the existence of positive feedback at the ensemble level that is not observed in the individual molecules (24). Finally, we developed a minimal kinetic model that incorporates details of the SOS_FL_ autoinhibition release mechanism, which successfully recapitulates the experimental observations. From these results, we conclude that the observed positive feedback in Ras activation by receptor-recruited SOS_FL_ is an ensemble effect stemming from RasGTP-mediated enhancement in the rate of autoinhibition release. Recently, several small-molecule inhibitors that target the SOS:Ras interaction have emerged as candidates for pan-KRas cancer therapy (29,30). Developing a comprehensive understanding of the mechanism underlying Ras activation by SOS may be useful for understanding the behavior SOS:Ras targeting drugs.

## Results

### The receptor-mediated recruitment of SOS_FL_ is not sensitive to Ras nucleotide state

Although the catalytic activity of SOS does not exhibit Ras-nucleotide state sensitivity at the single-molecule level (24), enhanced membrane recruitment provides an alternative mechanism for positive feedback. The SOS catalytic core (SOS_CAT_) and SOS_HDPC_ constructs, both of which lack the C-terminal PR domain responsible for receptor-mediated membrane recruitment (see SOS domain structure in Fig. 1), are primarily recruited to the membrane through binding Ras and exhibit preference for RasGTP. Recruitment of enzymes that act on membrane substrates to the membrane via their own product is a direct form of positive feedback (31,32). Increased recruitment to the RasGTP-containing membrane is a likely source for the observed positive feedback in SOS_CAT_ and SOS_HDPC_. The native SOS_FL_, however, is robustly autoinhibited—including with respect to Ras binding (17,18,22,33)—and is primarily recruited to the membrane via Grb2-mediated binding to phosphorylated receptor or scaffold proteins on the membrane (LAT in the experiments described here, as shown in Fig. 1). Thus, the native autoinhibition in SOS_FL_ is expected to prevent the recruitment-based positive feedback observed in truncated SOS constructs. To test this hypothesis, we perform detailed kinetic analysis of SOS recruitment in a supported bilayer configuration (22,23,24,25,34).

Purified Ras was tethered to the supported lipid bilayer through cysteine-maleimide chemistry at a density ∼2000 molecule per μm^2^, following established protocols (24). Ras was preincubated with unlabeled SOS_CAT_ to control the initial nucleotide state. Residual SOS_CAT_ was thoroughly washed away before initiating the recruitment assay. To ensure similar densities of proteins incubated on the membrane, flow chambers for RasGDP and RasGTP were prepared side by side. SOS constructs were labeled with Alexa Fluor 555 or Alexa Fluor 647 and the recruitment of SOS to the membrane was quantified using single-molecule counting via TIRF microscopy. Excess nucleotide was included in each flow chamber (GDP in the RasGDP chamber, and vice-versa for the RasGTP chamber) so that the nucleotide state of Ras would not change over the course of the measurement. In the SOS_FL_ recruitment experiment, the cytosolic domain of scaffold protein LAT (with N-terminal His10 tag (35)) was linked to the bilayer through nickel-histidine interaction to achieve experimental densities of ∼300–1000 molecules per μm^2^. LAT was maintained in a fully phosphorylated state by the promiscuous Src family kinase, Hck (with His6 tag), which was also on the bilayer (26). In solution, Grb2 was included in the system at 20 nM to enable recruitment of SOS_FL_ to the membrane through a LAT·Grb2·SOS_FL_ complex, which we refer to as receptor-mediated recruitment. The experiments were performed with physiological concentrations of LAT (in condensates) (36), Ras (as reported in Ras nanoclusters) (37), Grb2, and SOS (38). In reconstitution, fully percolated condensates that span the entire sample can be created (23,27), but the experiments described here were performed toward the dispersed end of this spectrum. Since SOS_FL_ alone can cross-link multiple Grb2·LAT, there is still microscopic LAT clustering and multivalent SOS engagement in these experiments—even while macroscopically extended condensates are not seen.

**Figure 2:**
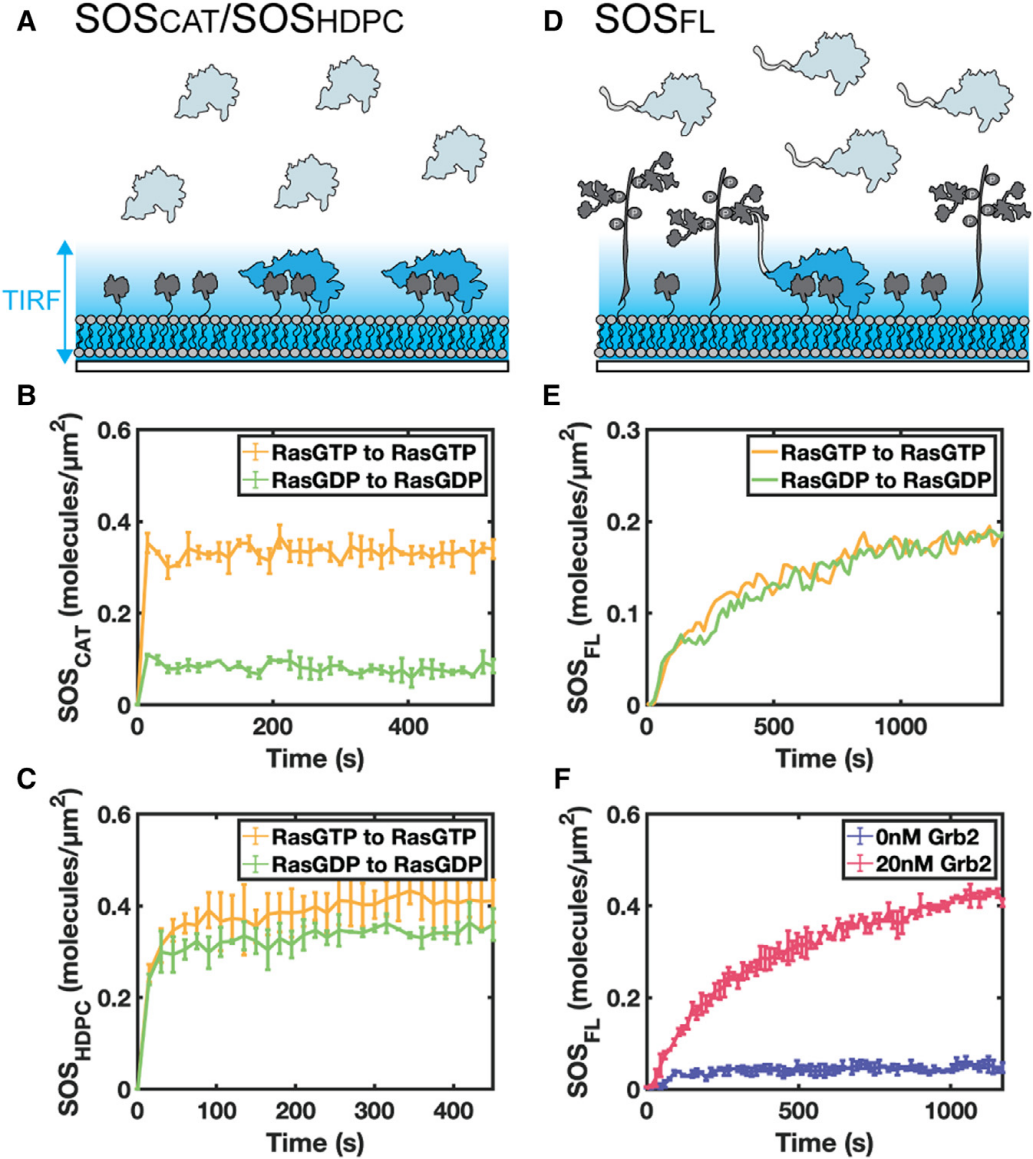
The receptor-mediated recruitment of SOS_FL_ is not sensitive to Ras nucleotide state. (*A*) Schematic of SOS_CAT_ or SOS_HDPC_ recruitment to Ras functionalized supported membrane. TIRF microscopy allows the quantification of SOS membrane recruitment under different membrane composition. Ras nucleotide-dependent membrane recruitment of (*B*) SOS_CAT_ (*n* = 2) and (*C*) SOS_HDPC_ (*n* = 2). (*B*) and (*C*) do not include LAT and Grb2. (*D*) Schematic of SOS_FL_ recruitment to pLAT and Ras functionalized supported membrane via Grb2. Both (*E*) and (*F*) include LAT and Grb2. (*E*) Ras nucleotide-independent membrane recruitment of SOS_FL_ (*n* = 1). (*F*) SOS_FL_ recruitment depends on Grb2 concentration (*n* = 2). Experiments on each plot were conducted on the same day to prevent significant fluctuations in membrane protein density. The error bars represent the standard deviation of the runs.

Measurements of Ras-mediated recruitment of Alexa647-labeled SOS_CAT_ and Alexa647-labeled SOS_HDPC_ to RasGTP or RasGDP functionalized membranes confirm a strong preference by SOS_CAT_ for RasGTP and a mild preference by SOS_HDPC_ (Fig. 2, *A–C*). Experiments at higher SOS_HDPC_ concentrations are shown in Fig. S1 *A*. Some of this observed Ras-nucleotide preference likely stems from the binding affinity differences between SOS-RasGTP and SOS-RasGDP at equilibrium (18,19). However, single-molecule SOS studies have revealed that SOS has several internal states, whereby a subset of membrane recruited molecules become stably associated with the membrane and enter a processive, catalytically active state while others exhibit only transient membrane interactions (22,34). Thus, the ratio between processive and transient states of SOS will also affect equilibrium density differentially between different SOS constructs (34). The discrepancy in the extent of Ras-nucleotide preference between SOS_CAT_ and SOS_HDPC_ may also stem from the accessibility of Ras binding site, as the regulatory domains keep SOS_HDPC_ in a more autoinhibited conformation than SOS_CAT_ (18). In addition, PI(4,5)P_2_ and PH domain interaction (33,39) may enhance SOS_HDPC_ membrane association in a Ras-independent manner.

In contrast to SOS_CAT_ and SOS_HDPC_, SOS_FL_ clearly exhibits Ras nucleotide-independent recruitment to the membrane (Fig. 2, *D* and *E*). Another experiment at a higher SOS_FL_ concentration is shown in Fig. S1 *B*. SOS_FL_ is primarily recruited via Grb2 to phosphorylated LAT on the membrane and exhibits minimal membrane binding in the absence of Grb2 (Fig. 2 *F*). In addition, there was no measurable effect in the initial SOS recruitment kinetics due to Ras density (Fig. S2). From the SOS_FL_ membrane recruitment data in Fig. 2 *E*, it is evident that both the rise time (reflective of the kinetic on-rate for membrane recruitment) and the plateau density (reflective of equilibrium) are independent of Ras nucleotide state. This also directly shows that the dissociation rate, or kinetic off-rate, of SOS_FL_ is insensitive to the Ras nucleotide state as well. Therefore, even though the SOS_FL_ allosteric site has a higher binding affinity for RasGTP, this experimental evidence indicates that such preference does not contribute significantly to SOS_FL_ membrane recruitment. Thus, SOS_FL_ does not exhibit positive feedback in receptor-mediated membrane recruitment.

### Ensemble catalytic activity of SOS_FL_ exhibits RasGTP-driven positive feedback

**Figure 3:**
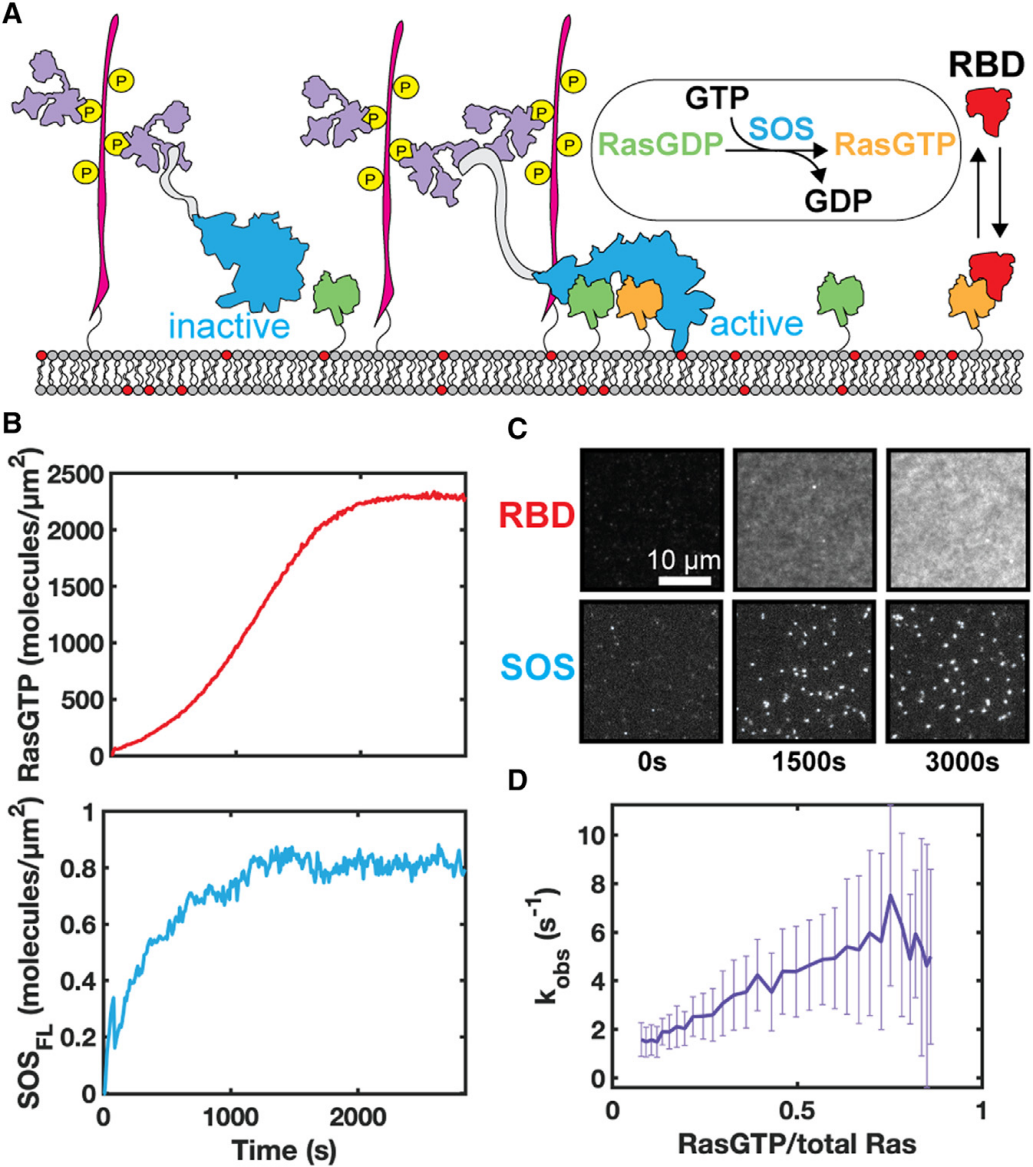
SOS_FL_ autoinhibition release depends on RasGTP levels. (*A*) Schematic of the activity assay for determining the fraction of active SOS on the membrane. Recruited SOS_FL_ is either in the process of autoinhibition release or in the active conformation. RBD sensor acts as a real-time RasGTP level readout. (*B*) Ras activation inferred from RBD recruitment and SOS membrane density were measured simultaneously. (*C*) Example time-lapse images for RBD and SOS recruitment. (*D*) Ensemble average catalytic rate (k_obs_) for all SOS_FL_ recruited to the membrane at different RasGTP level. For the k_obs_ calculation, the RasGTP level and SOS_FL_ membrane density were averaged every five time points, and the standard deviation was calculated and propagated as the error, represented by the error bar.

Ras activation studies by SOS_FL_ were performed similarly as the recruitment assays discussed above, except that we simultaneously measured SOS_FL_-catalyzed Ras nucleotide exchange from RasGDP to RasGTP (Fig. 3 *A*). RasGTP density was measured by the membrane recruitment of a fluorescently labeled RBD sensor, adapted from Raf with K65E point mutation to ensure fast binding and unbinding kinetics to Ras (23). The RBD sensor provides a real-time readout of RasGTP levels in the system, and was calibrated to directly reflect RasGTP density as measured by fluorescence correlation spectroscopy in Fig. S3. When the RBD sensor intensity reaches a plateau, the readout indicates the total Ras level, as all the RasGDP has been converted to RasGTP. This defines the RasGTP/total Ras ratio. RasGTP levels and SOS_FL_ membrane density (Fig. 3 *B*) were simultaneously measured. Example time-lapse images for RBD and SOS_FL_ membrane recruitment are shown in Fig. 3 *C*.

Ras densities are significantly higher than SOS_FL_ densities in these experiments and SOS_FL_ is working under substrate saturating conditions (24). Under these conditions, the overall rate of RasGTP production is first order with SOS density and proportional to the overall SOS turnover rate by the ratio of RasGDP/Ras_total_. This proportionality results from the fact that, even when the catalytic site of SOS binds RasGTP, it still spends an entire enzymatic cycle to exchange GTP for GTP, with no net contribution to the overall RasGTP production rate. The following rate equation was used to calculate the per-molecule catalytic rate of SOS_FL_:

(1) $$\frac{\text{dRasGTP}}{\text{dt}}=k_{\text{obs}}\times \sigma \left[\text{SOS}\right]\times \sigma \left[\text{RasGDP}\right]/\sigma \left[{\text{Ras}}_{\text{total}}\right]$$

where k_obs_ is the ensemble average catalytic turnover per SOS_FL_ molecule, σ denotes surface density. The increasing apparent per-molecule catalytic rate (Fig. 3 *D*) shows that SOS_FL_ exhibits RasGTP-dependent positive feedback, but evidently not by the same mechanism as SOS_CAT_ and SOS_HDPC_.

With membrane recruitment and molecular catalytic activity already eliminated as possible mechanisms for SOS_FL_ positive feedback, the only remaining mechanistic possibility is the autoinhibition release process. SOS_FL_ is recruited to the membrane in an autoinhibited state (here referred to as SOS_0_ for kinetic modeling purposes). After initial membrane recruitment, the simplest model for the autoinhibition release process involves one dominant rate-limiting kinetic intermediate (SOS_1_) before entering its active state (SOS_p_), during which SOS begins to processively catalyze nucleotide exchange in Ras (23). If RasGTP levels on the membrane alter the kinetics of SOS progression to its activated state, then this provides an alternative mechanism for RasGTP-driven positive feedback. We examine this possibility quantitatively with kinetic modeling.

**Figure 4:**
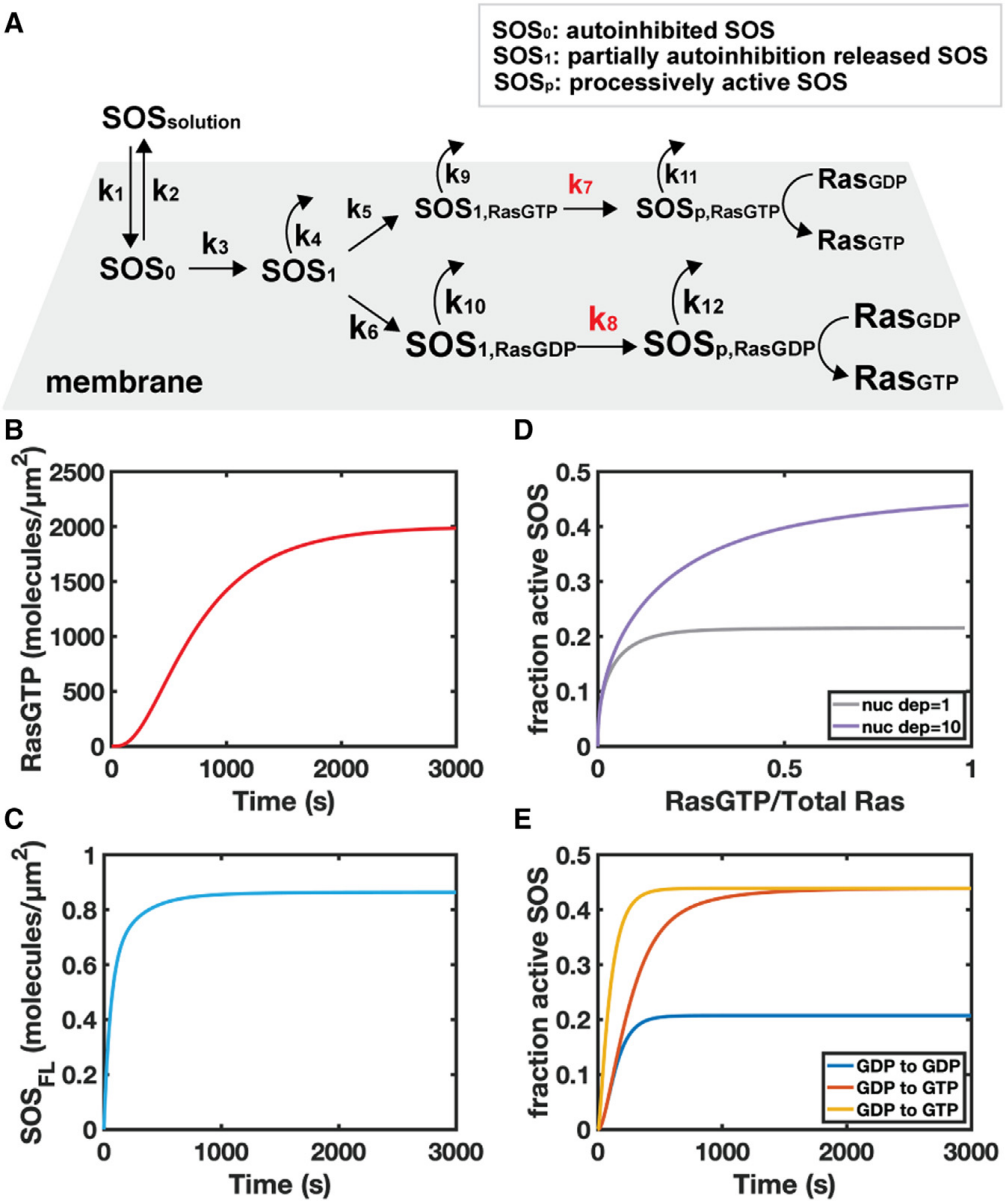
Kinetic simulations show accelerated autoinhibition release as a mechanism for positive feedback in SOS activation. (*A*) Schematic for the kinetic model. (*B*) Plot of RasGTP level over time in RasGDP to RasGTP nucleotide exchange reaction from simulation. (*C*) Plot of SOS membrane recruitment over time, obtained simultaneously with Ras activity. (*D*) Plot of fraction of active SOS over time, obtained from the ratio between processively active SOS and total SOS on the membrane. (*E*) Plots of Ras nucleotide-dependent activation of SOS.

The proposed kinetic scheme is depicted in Fig. 4 *A*. SOS_FL_ recruitment and progression to its kinetic intermediate (SOS_1_) state are represented as Ras density independent. SOS_1_ then binds RasGTP or RasGDP at the allosteric binding site and the final transition to SOS_p_ has different rates depending on the nucleotide state of allosterically bound Ras. This is the presumed sequence since PI(4,5)P_2_ binding releases the DH-PH domain and exposes the Ras allosteric binding pocket (33,40). All membrane-bound SOS molecules may dissociate back into the solution with rates that depend on their activity state. The relaxation time back to the autoinhibited state once a SOS molecule has desorbed is short compared with the recycling time of SOS_FL_ back to the membrane. Therefore, all the newly bound SOS molecules start in an autoinhibited conformation. The choice to model SOS activation as a one-way process builds upon a previous study of ours (41), in which we confirmed that allowing reversibility in the activation intermediate steps does not qualitatively change SOS activation behavior. In addition, under physiological conditions, the SOS activation process occurs out of equilibrium with a net flux toward activation. The rate equations for each membrane-bound species are listed in the supporting information. The temporal evolution of the system was numerically solved as differential equations in MATLAB.

Based on the proposed model, there are three potential steps giving rise to positive feedback: these include Ras binding to the SOS allosteric site (k_5_ and k_6_), Ras-bound SOS entering the processively active state (k_7_ and k_8_), and Ras-bound SOS desorbing from the membrane (k_9_, k_10_, k_11_, and k_12_). Since we inferred from the SOS_FL_ receptor-mediated recruitment assay that the membrane dissociation rate of SOS_FL_ is insensitive to the Ras nucleotide state (Fig. 2 *E*), we focused on the first two mechanisms in the simulation.

Ras binding to the SOS allosteric site at equilibrium has been the subject of numerous studies investigating the details of positive feedback in Ras activation by SOS (18,19,20). However, the direct measurement of the kinetics of this binding process (k_5_ and k_6_) is lacking. We utilized the kinetics of SOS_CAT_ membrane recruitment obtained in the previous section to estimate the range of possible rates for SOS_1_ binding to Ras on the membrane (details shown in the supporting material). On both RasGTP and RasGDP bilayers, SOS_CAT_ reached a steady state in less than 15 s (Fig. 2 *B*). The binding rates to RasGTP and RasGDP were adjusted proportionally based on the difference in binding affinity of SOS_CAT_ (18). Although membrane adsorption kinetics are not directly transferable to collision rates on the membrane, this estimation reveals that the Ras binding step is a rapid process, significantly shorter than the time scale required for SOS activation (23). Consequently, we concluded that Ras binding is not a rate-limiting step in SOS activation. The transition rates of Ras-bound SOS into the processively active state (k_7_ and k_8_) have also not been experimentally measured before. Nevertheless, single-molecule measurements (23) indicate that the time scale for membrane recruited SOS_FL_ to reach the active state is on the order of tens of seconds.

Other kinetic parameters were derived from previous experimental measurements and adjusted to align with observed experimental data. The rate of solution SOS binding to the membrane (k_1_) was estimated using the early time points from our recruitment traces (Fig. S5). The rate of membrane dissociation of inactive SOS molecules (SOS_0_, SOS_1_, and SOS_1,RasGXP_) were all treated to be equal to a rate (k_off_transient_) that was measured from a previous experimental study (23). While improvements in these parameters by considering additional membrane interactions would likely lead to more accurate simulations, we have opted not to extend the simulation details beyond the level of available experimental input for this model. The membrane dissociation rates of active SOS_FL_ molecules (SOS_p, RasGXP_), k_off_processive_, were estimated from single-molecule activation profiles (34).

After adjusting the parameters (details in the supporting material), we introduced a nucleotide preference to k_7_ and k_8_ (k_7_ = 10× k^8^). The simulation results confirm that accelerated autoinhibition release can indeed serve as a mechanism for positive feedback in activation of Ras by SOS. Firstly, the nucleotide exchange reactions followed a time scale similar to that observed in experiments, and the overall SOS recruitment reached a similar level (Fig. 4, *B* and *C*). Secondly, the fraction of active SOS, which is directly proportional to the apparent average catalytic rate per SOS molecule (see supporting material for details), increased with higher RasGTP levels. RasGTP-enhanced SOS activation is not observed when the nucleotide preference is set to 1 (Fig. 4 *D*). Moreover, the simulation allowed us to calculate the fraction of active SOS during homonucleotide exchange (RasGTP to RasGTP and RasGDP to RasGDP; Fig. 4 *E*), which could not be experimentally measured. The simulation results revealed that the system reached a steady state with a higher fraction of active SOS when cycling RasGTP to RasGTP since all SOS activations occurred through the accelerated pathway. In contrast, in the RasGDP homonucleotide exchange condition (RasGDP to RasGDP), the fraction of activated SOS remained low throughout the simulation. A shift from a low fraction of active SOS to a high fraction was observed in the RasGDP to RasGTP simulation as more RasGTP was produced from the nucleotide exchange reaction, resembling the positive feedback seen in the Ras activation by SOS_FL_ experiments.

## Discussion and conclusion

In this study, we have resolved a distinct mechanistic source of positive feedback in the native SOS_FL_ protein that contrasts behavior of its truncated forms. SOS has long been known to play an important role in the bimodal behavior of signaling through Ras and the MAPK pathway (4,5). Although SOS has a preference toward RasGTP binding at the allosteric site (18), which would seem to be the most obvious source of positive feedback in its Ras activation kinetics, this preference becomes inconsequential in the native SOS_FL_ due to its autoinhibition. In addition, single-molecule experiments have demonstrated that SOS catalytic activity is independent of Ras-nucleotide state at the molecular level—ruling this out as a positive feedback mechanism. Here, we have shown, through both experiments and kinetic modeling, that it is the autoinhibition release process in SOS_FL_ that provides the dominant source of positive feedback.

A few other recent studies have also revisited the origin of the positive feedback in Ras activation by SOS. The studies by Vo et al. (19) and Liao et al. (20) focused on the interaction between Ras and SOS catalytic site and allosteric site, suggesting that RasGDP is a weaker binder than RasGTP toward the allosteric site, but a stronger binder toward the catalytic site. However, preferential binding of RasGDP to the catalytic site is not likely a driver of the positive feedback observed in the experiments described here, which were all performed at high Ras density where the catalytic nucleotide exchange step—not the RasGDP binding to the catalytic site—is rate limiting.

Another paper from our own group (34) has examined reconstituted bimodality in a competitive Ras activation-deactivation assay. In that work, processivity of SOS was further identified as a key driver of bimodality in the competitive reaction. Taken together, all these recent studies and the present work reveal a multifaceted mechanism of positive feedback in the activation of Ras by SOS. Although simple effects of allosteric activation by preferential binding of RasGTP to the allosteric binding site clearly leads to positive feedback in truncated SOS variants, the SOS_FL_ protein is much more complex and relies heavily on autoinhibition release and processivity to achieve its macroscopic behavior.

## Author contributions

H.R., A.A.L., and J.T.G. designed the study. H.R., A.A.L., L.J.N.L., and J.B.D. prepared reagent. H.R. performed experimental data collection. H.R. performed kinetic modeling. H.R. analyzed data. H.R. and J.T.G. wrote the manuscript. All authors edited and approved the manuscript.

## Supporting material

Document S1. Figures S1–S9

Document S2. Article plus supporting material
