# Supplementary material for: Positive feedback in Ras activation by full-length SOS arises from autoinhibition release mechanism
Source: Biophys J. 2024 Jul 16;123(19):3295–303. doi: 10.1016/j.bpj.2024.07.014 (PMC11480760; doi:10.1016/j.bpj.2024.07.014)
Supplement: Document S1. Figures S1–S9 [file mmc1.pdf]

Biophysical Journal, Volume *123*

Supplemental information

Positive feedback in Ras activation by full-length SOS arises from auto-inhibition release mechanism

He Ren, Albert A. Lee, L.J. Nugent Lew, Joseph B. DeGrandchamp, and Jay T. Groves

Supplementary Information for "Positive feedback in Ras activation by full-length SOS arises from autoinhibition release mechanism"

Authors
He Ren[1], Albert A. Lee[2,3], L.J. Nugent Lew[1], Joseph B. DeGrandchamp[1,4], Jay T. Groves[1,5,*]

Affiliations
[1]Department of Chemistry, University of California Berkeley, Berkeley, CA 94720, USA
[2]Department of Molecular and Cell Biology, University of California Berkeley, Berkeley, CA 94720, USA
[3]Current address: Department of Biochemistry, Stanford University, CA 94305, USA
[4]Current address: Institute for Digital Molecular Analytics and Science, Nanyang Technological University, 639798 Singapore.
[5]Division of Molecular Biophysics and Integrated Bioimaging, Lawrence Berkeley National Laboratory, Berkeley, CA 94720, USA.

*Correspondence: jtgroves@lbl.gov

Materials and Methods

Protein purification

LAT-His10 Human LAT cytosolic domain (27-233) was purified with N-terminal 10-Histidine tag (1). The histidine tag was uncleaved for lipid bilayer functionalization.
HCK-His6 Human full length HCK was purified with N-terminal 6-Histindine tag and a TEV site in BL21 transformed with YopH as described in (2). The histidine tag was uncleaved for lipid bilayer functionalization.
SOS$_{FL}$ Human SOS1 was purified though split intein as described in (3).
SOS$_{HDPC}$ The purification of human SOS1 HDPC (1-1049) was described in (4).
SOS$_{CAT}$ The purification of human SOS1 catalytic core (566-1049) was described in (4).
Grb2 Human full length Grb2 was purified with N-terminal 6-Histindine tag and a TEV site as described in (5).
Ras Human H-Ras (1-181, C118S) was purified with N-terminal 6-Histindine tag and a TEV site as described in (3).
RBD sensor RBD-K65E was derived from human Raf1 Ras binding domain with one point mutation (56-131, K65E). It was purified with N-terminal 6-Histine tag, GST, a PreScission site, and a SNAP tag as described in (3).

Protein Labeling

LAT, SOS$_{FL}$, and SOS$_{HDPC}$ were labeled with Alexa-555 or Alexa-647 C2 Maleimide (Invitrogen). Proteins were diluted to 100 μM or less with 5mM TCEP (Sigma-Aldrich), then react with 1 mM maleimide dye for 2 hr in room temperature. The reaction was then quenched with 5 mM 2-mercaptoethanol (BME, Sigma-Aldrich) for 10 min. Excessive dyes were removed by size exclusion chromatography (SEC) via FPLC (Superdex 75, Cytiva).

RBD sensor was labeled with SNAP-Surface Alexa-488 or Alexa-647 (New England Biolabs). Proteins were mixed with the SNAP-Surface dye at 1:1.5 reaction in PBS and 10 mM DTT at 18 °C overnight. The reaction mixture was buffer exchanged to 20 mM Tris, 200 mM NaCl, 10% glycerol, and 1 mM TCEP before running though SEC to remove excess dye.

Supported lipid bilayer preparation and assay

SUV Small unilamellar vesicles were made using 1,2-dioleoyl-sn-glycero-3-phosphocholine (DOPC), L-α-phosphatidylinositol-4,5-bisphosphate (Brain, Porcine) (ammonium salt) (PI(4,5)P_2), 1,2-dioleoyl-sn-glycero-3-phosphoethanolamine-N-[4-(p-maleimidomethyl)cyclohexane-carboxamide] (sodium salt) (MCC), and 1,2-dioleoyl-sn-glycero-3-[(N-(5-amino-1-carboxypentyl)iminodiacetic acid)succinyl] (nickel salt) (Ni-NTA) from Avanti Polar Lipids. Lipids were mixed in an etched and dried round bottom flask to yield 2 mmol total lipid at 94:2:2:2 (mol%) DOPC:PI(4,5)P_2:MCC:Ni-NTA. After mixing, lipids were dried by rotovap in a 37°C water bath for 2 min then in room temperature for 15 min. Later, the round bottom flask was flushed with N_2 for 15 min. The lipid film was hydrated with 2 mL of milli-Q water for a final concentration of 1 mM total lipids. The cloudy lipid solution was added to a 5 mL polypropylene tube and treated by 3 mm stepped tip sonication at 33% amplitude, 20 s sonication, 50 s rest, 5 cycles (Sonics VCX750) while submerged in ice. The lipid solution was clarified after sonication.

Reaction chamber Precision Schott D 263 M glass coverslips (#1.5H, 75 x 25 mm) was rinsed with milli-Q. Coverslips were then etched with piranha made with 3:1 ratio of concentrated H_2SO_4 (Fisher Scientific) and 30% H_2O_2 (Fisher Scientific) for 5 min and rinse extensively with milli-Q water. To make the experiment chamber, etched coverslips were blow-dried with N_2 and attached to 6-channel flow cells (sticky-Slide VI 0.4, ibidi).

SLB Supported lipid bilayer were made through SUV vesicle fusion. The 1mM lipid solution was diluted to 0.25 mM in PBS (Fisher Scientific). Then, 150 µL of 0.25 mM lipid solution was added to each channel and incubate for 30 min at room temperature. After incubation, the channel was washed with 1mL PBS and blocked with 1mg/mL blocker casein (Thermo Fisher Scientific) in PBS for 10 min. Each channel was washed with 1mL PBS.

Ras bilayer To attach H-Ras on the bilayer, 100 µL of 0.3-0.5 mg/ml H-Ras in reaction buffer (40 mM HEPES (pH 7.4), 100 mM NaCl, 5 mM $MgCl_2$) was added to each channel at room temperature for 2 hr 30 min. Each channel was washed with 1mL PBS and flowed with 100 µL of 5 mM BME in reaction buffer to quench the remaining free maleimide. Each channel was washed with 1 mL PBS again before the Ras bilayer being stored in 100uL of 100 µM guanosine-5'-diphosphate disodium salt (Chem-Impex) in reaction buffer overnight at 4 °C.

LAT bilayer To attach LAT and phosphorylate LAT on the bilayer, the chamber was first taken out of 4 °C, equilibrated to room temperature, and washed with 1mL PBS. Then, 100 µL of 14 nM of LAT and 12.5 nM of HCK were added to each channel for 40 min. After washing the channel with 1 mL PBS, 100 µL of 1 mM of ATP and 100 µM of GDP were added to the channel for LAT phosphorylation and ensure nucleotide loading. Due to the variations in SUV composition, including Ni-NTA lipid composition, LAT densities

vary from day to day, and the variation is essentially unavoidable. Experiments conducted on the same day using the same batch of SUVs usually exhibit minimal variation in LAT density. Therefore, we only compare recruitment traces obtained in the same day.

RasGXP recruitment experiments Due to the intrinsic activity of Ras and supplied GDP in the solution, Ras started as RasGDP on the bilayer. To make RasGTP bilayer, the channel was washed with 1 mL PBS, then 100 µL of 2 nM SOS_{cat}, 1 mM of GTP (Guanosine-5′-Triphosphate Trisodium Salt, Fisher Scientific), and 1 mM of ATP in reaction buffer was flowed into each channel for 30 min. To control for the perturbation brought by SOS_{cat} to the experiment, a RasGDP bilayer was prepared side by side with 100 µL of 2nM SOS_{cat}, 1 mM of GDP, and 1mM of ATP in reaction buffer for 30 min. To wash away SOS_{cat} in the system, 3 mL of PBS with 100 µM GTP or 100 µM GDP was flow through the RasGTP bilayer or RasGDP bilayer respectively. The channel was stored in 1 mM of ATP and 100 µM of GTP or GDP in reaction buffer before any assay. 500 pM of Alexa647-labelled SOS_{CAT}, SOS_{HDPC}, or SOS_{FL} (and 20nM Grb2 for SOS_{FL} exp) was added with 20 nM of Alexa488-labelled RBD, and 1 mM GXP (GTP for RasGTP membrane, GDP for RasGDP membrane) were injected in the flow cell.

Imaging buffer All imaging experiments were ran in imaging buffer with 40 mM HEPES (pH 7.4), 100 mM NaCl, 5 mM MgCl2, 10 mM BME, 100 µM ATP, and 2 mM UV-treated Trolox.

Microscope hardware and imaging acquisition

TIRF imaging experiments were performed on an inverted Nikon Eclipse Ti microscope using a 100X Nikon oil-immersion TIRF objective (1.49 NA), and the 1.5X lens tube were also used in the experiment. 488/561/647 nm diode laser (OBIS laser diode, Coherent) were controlled by a custom built Solemere laser driver to excite fluorophores, and the images were acquired with an iXon Ultra 897 EMCCD camera (Andor Technology). All microscope hardware was controlled using Micro-Manager v4.0. Images were acquired with 15 s time lapse, 500 EM gain, at 100 ms exposure time. High laser intensity (20 mW out of box) was used for single molecule density experiments, and lower laser intensity (2mW) was used for bulk density measurements. For all kinetic traces, middle 250*250 pixels were used to extract data. RBD fluorescence signal were corrected for uneven illumination using shading corrector (Fiji). Single particles were identified with TrackMate (Fiji).

Ras density and LAT density titration

Surface density of Ras and LAT were measured using fluorescence correlation spectroscopy (FCS). The setup was previously described in (6). The FCS imaging experiments were performed on a home-built confocal system on a Nikon TE2000 inverted microscope using a 100x Nikon oil-immersion PlanFluor objective (1.30 NA). A pulsed supercontinuum laser (SuperK Extreme; NKT Photonics) with 100 ps pulse duration at 19.5 MHz repetition rate was used. The signals were recorded by an avalanche photodiode detector (Hamamatsu) and then processed by a hardware correlator (Correlator.com). Then, the autocorrelation function was fitted to a 2D diffusion model to extract surface density and diffusion coefficient. Ras density was measured with Ras nucleotide exchange to EDA-GDP-ATTO-488 (Jena Bioscience), as

described in (6). LAT density was measured with Alexa-555 labeled LAT and corrected for labeling efficiency. A calibration was developed from Ras density measured from FCS and Ras density measured by RBD TIRF signal. Similar LAT density calibration was developed from FCS and TIRF fluorescently labeled LAT signal.

Kinetic simulation

The reaction pathway was listed in Fig. 4A. Since the nucleotide exchange rate (k_{SOS}) of processive SOS_{FL} is nucleotide independent, the fraction of active SOS_{FL} (f(act)) is proportional to the ensemble average catalytic rate:

$$k_{obs} = \frac{\sigma[SOS_{active}]\times k_{SOS} + \sigma[SOS_{inactive}]\times 0}{\sigma[SOS_{active}]+\sigma[SOS_{inactive}]} = \frac{\sigma[SOS_{active}]}{\sigma[SOS_{active}]+\sigma[SOS_{inactive}]}\times k_{SOS} = f(act)\times k_{SOS}$$

Rate equation

The time evolution for the number of each species was solved numerically (ode45 for 3000s) in MATLAB_R2020B.

1. dy(1)/dt=d[SOS_0]/dt=k1*[SOS_solution]-k2*[SOS_0]-k3*[SOS_0]
2. dy(2)/dt=d[SOS_1]/dt= k3*[SOS_0]-k4*[SOS_1]-k5*[RasGTP] *[SOS_1]-k6*[RasGDP] *[SOS_1]
3. dy(3)/dt=d[SOS_1_RasGTP]/dt= k5*[RasGTP] *[SOS_1]-k9*[SOS_1_RasGTP]-k7*[SOS_1_RasGTP]
4. dy(4)/dt=d[SOS_1_RasGDP]/dt= k6*[RasGDP] *[SOS_1]-k10*[SOS_1_RasGDP]- k8*[SOS_1_RasGDP]
5. dy(5)/dt=d[SOS_p_RasGTP]/dt= k7*[SOS_1_RasGTP]-k11*[SOS_p_RasGTP]
6. dy(6)/dt=d[SOS_p_RasGDP]/dt= k8*[SOS_1_RasGDP]-k12*[SOS_p_RasGDP]
7. dy(7)/dt=d[RasGTP]/dt=kcat*([SOS_p_RasGTP]+[SOS_p_RasGDP])*[RasGDP]/[Ras_total]

The following kinetic parameters were used. In next section, the process of how we got the parameters is described.

k1 = k_{on}
k2 = $k_{off\_transient}$
k3 = k_{01}
k4 = $k_{off\_transient}$
k5= $k_{ras\_binding}$ * 6
k6 = $k_{ras\_binding}$
k7 = k_{1p} * **nucleotide dependence**
k8 = k_{1p}
k9 = $k_{off\_transient}$
k10 = $k_{off\_transient}$
k11 = $k_{off\_processive}$
k12 = $k_{off\_processive}$

[SOS_solution]=0.15 nM
[Ras_total]= 2000 μm^{-2}
k_{on} = 0.077 s^{-1} nM^{-1} μm^{-2}
nucleotide dependence = 10
$k_{off\_transient}$ = 0.016 s^{-1}
$k_{off\_processive}$ = 0.01 s^{-1}
k_{01} = 0.01 s^{-1}
$k_{ras\_binding}$ = kon'/ [Ras_total] = 0.3/2000 $s^{-1}molecule^{-1}\mu m^{2}$ (kon' definition see next section)
k_{1p} = 0.01 s^{-1}
k_{cat} = 10 s^{-1}

[SOS_solution] adjustment
The approximated k_{on} from Figure S5 is 0.077*[SOS_solution] $s^{-1}nM^{-1}\mu m^{-2}$, but the binding frequency measurement is specific to the LAT density which varies by each experimental realization. Therefore, we adjusted the [SOS_solution] in our simulations in order to match the SOS membrane recruitment level as the experiment results shown in Figure S6.

$k_{ras\_binding}$ estimation
We approximated $k_{ras\_binding}$ using SOS_{CAT} membrane adsorption kinetics. If assuming SOS_{CAT} membrane binding follows a simple kinetic process as:

SOS + Ras $\xrightarrow{\text{kon}}$ SOS*Ras

SOS*Ras $\xrightarrow{\text{koff}}$ SOS + Ras

By conservation of mass, SOS*Ras + SOS = SOS_total.

$$\frac{\mathrm{d[SOS * Ras]}}{\mathrm{dt}} = \mathrm{kon} \cdot [\mathrm{SOS}] \cdot [\mathrm{Ras}] - \mathrm{koff} \cdot [\mathrm{SOS} * \mathrm{Ras}]$$

Because Ras density is significantly higher than SOS density on the membrane, we can assume:

$$[\mathrm{Ras}] = [\mathrm{Ras_{total}}] - [\mathrm{SOS} * \mathrm{Ras}] \approx [\mathrm{Ras_{total}}]$$

Then the equation can be written as:

$$\mathrm{kon}' \approx \mathrm{kon} \cdot [\mathrm{Ras_{total}}]$$

$$\begin{aligned}\frac{\mathrm{d[SOS * Ras]}}{\mathrm{dt}} &= \mathrm{kon}' \cdot [\mathrm{SOS}] - \mathrm{koff} \cdot [\mathrm{SOS} * \mathrm{Ras}] \\ &= \mathrm{kon}' \cdot (\mathrm{SOS_{total}} - [\mathrm{SOS} * \mathrm{Ras}]) - \mathrm{koff} \cdot [\mathrm{SOS} * \mathrm{Ras}] \\ &= \mathrm{kon}' \cdot \mathrm{SOS_{total}} - \mathrm{kon}' \cdot [\mathrm{SOS} * \mathrm{Ras}] - \mathrm{koff} \cdot [\mathrm{SOS} * \mathrm{Ras}] \\ &= constant - (\mathrm{kon}' + \mathrm{koff}) \cdot [\mathrm{SOS} * \mathrm{Ras}]\end{aligned}$$

$$[\mathrm{SOS} * \mathrm{Ras}] = constant * (1 - e^{-(\mathrm{kon}' + \mathrm{koff})t})$$

For both RasGTP and RasGDP, SOScat reaches equilibrium before 15s. We can fit the above equation to t=15, normalized SOScat recruitment = $\frac{[\mathrm{SOS*Ras}]}{constant}$ =0.9999. Then $\mathrm{kon}' + \mathrm{koff}$ is around 0.6 s^{-1}. Since SOScat binding affinity (K_D) toward both RasGTP and RasGDP are in the micromolar range (7,8) in solution, we expect kon to be bigger than koff on membrane. Therefore, we varied kon' from 0.3, 3, to 30 and found that

because Ras binding is not a rate limiting step, there is very little difference between 0.3, 3, and 30, shown in Figure S7.

Additionally, to differentiate the RasGTP and RasGDP binding pathway, we varied the rate according to the difference in binding affinity of RasGTP (K_D ~3.6 μM) and RasGDP (K_D ~24.5 μM) to SOScat allosteric binding pocket (7). By assuming the difference in binding affinities primarily presents in the on rate, we concluded k5=6*k6.

k_{01} and k_{1p} estimation

Earlier single molecular measurements (3) have shown that the timescale for SOS getting into the active state is tens of seconds. k_{01} and k_{1p} determine the rate of SOS activation and the timescale for the bulk nucleotide reaction to be done. For simplicity, we define $k_{01}=k_{1p}$. Then, we varied k_{01} and k_{1p} from 0.05, 0.02, 0.01, and 0.005, and found 0.01 best reproduced experimental results, shown in Figure S8.

k_{cat} estimation

We estimated kcat to be 10 s^{-1} based on Figure 3D.

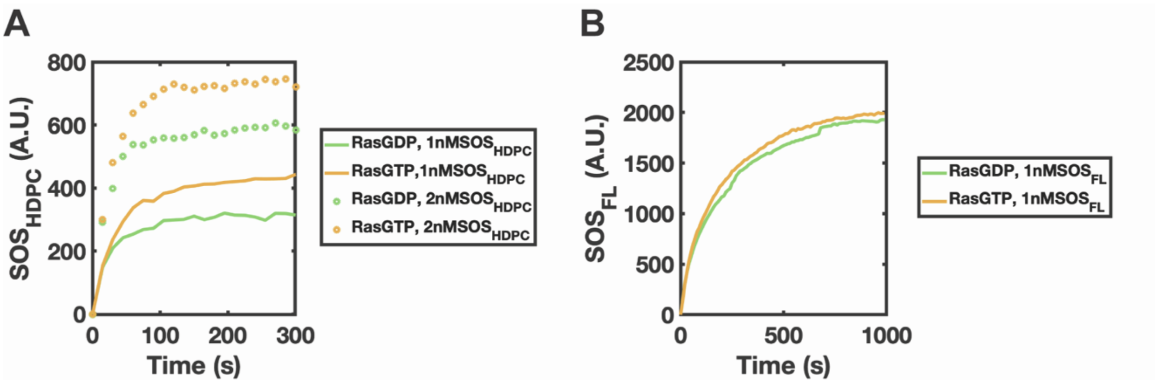

Figure S1. Additional SOS recruitment traces. Consistent with Figure 2, (A) SOS$_{HDPC}$ membrane recruitment is Ras nucleotide dependent (N=1). (B) LAT:Grb2 mediated SOS$_{FL}$ membrane recruitment is not Ras nucleotide dependent (N=1). Due to the high amount of SOS membrane recruitment, SOS density cannot be resolved by single particle counting in these experiments. Bulk fluorescence intensity, which is proportional to particle density, is shown in the y axis.

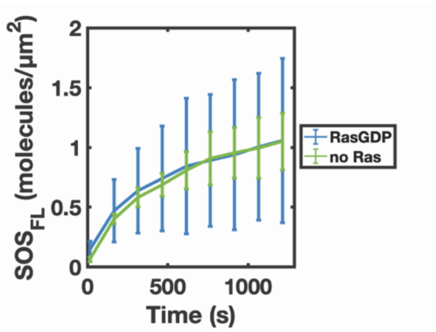

Figure S2. The initial SOS$_{FL}$ recruitment kinetics is independent of Ras density. We did not observe measurable effects in the initial SOS recruitment kinetics caused by the presence of Ras (N=2). Since Ras binding is involved in SOS ultimately reaching its catalytically active state, there is an intrinsic difference in the overall state of membrane-recruited SOS with or without membrane Ras. This naturally suggests that, in equilibrium, the overall amount of membrane recruited SOS will depend on Ras. The SOS$_{FL}$ membrane recruitment experiments, and likely the physiological system as well, however, do not examine equilibrium conditions. Instead, the results are driven by kinetics of the initial recruitment process, and these are dominated by Grb2.

(A)

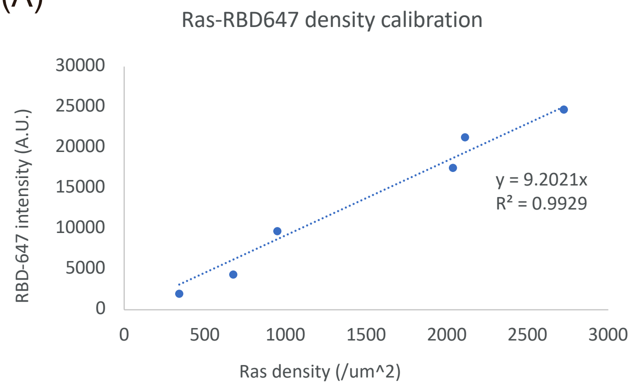

(B)

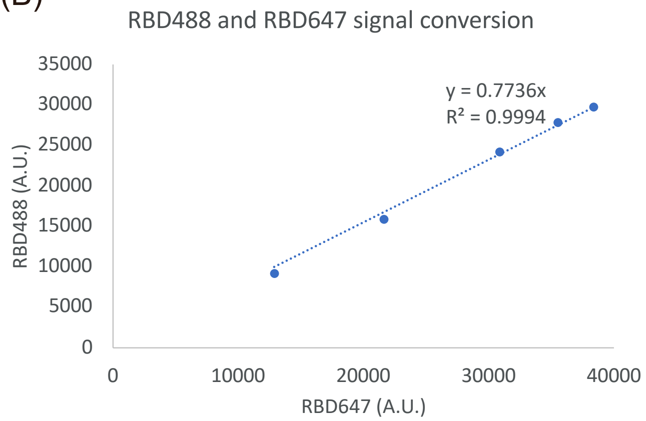

Figure S3. Ras density and RBD sensor calibration. (A) Ras density was measured by FCS using Ras with fluorescently labeled nucleotides. The same Ras bilayer was measured with RBD-Ax647, and the intensity was obtained from TIRFM. (B) RBD-Ax647 and RBD-Ax488 were added to the Ras bilayer simultaneously, and both signal intensities were measured with TIRFM.

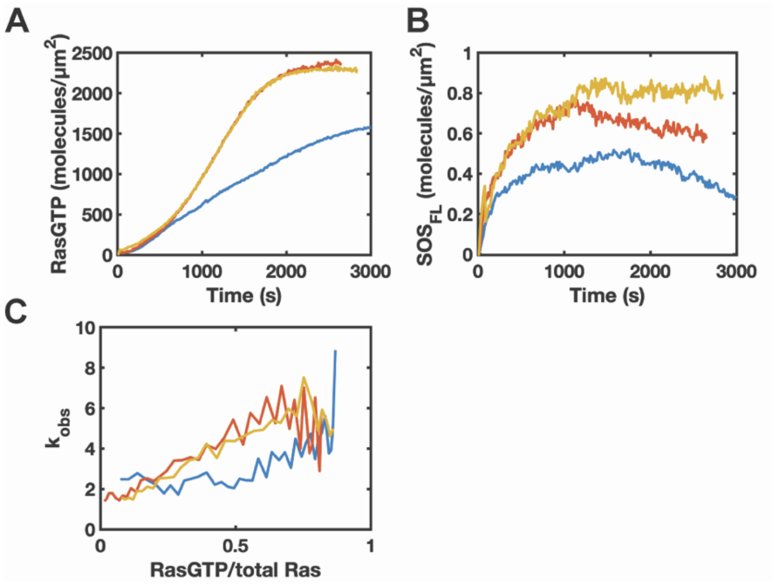

Figure S4. Ras activation and SOS membrane recruitment heterogeneity. Three representative traces for Ras activation (A), SOS membrane recruitment (B), and k_{obs} calculation.

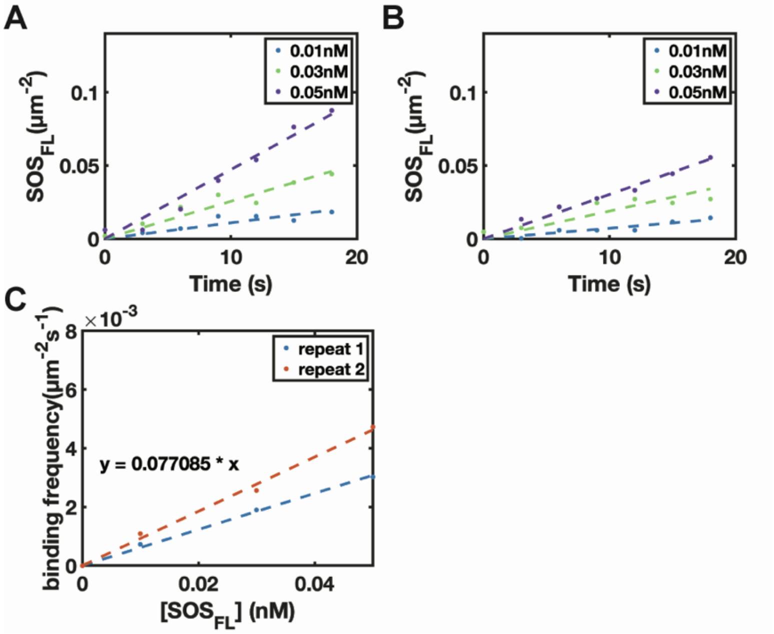

Figure S5. SOS_{FL} membrane recruitment rate estimation on a Ras bilayer with LAT and Grb2. Two repeats (A and B) for SOS_{FL} membrane recruitment at early time points. (C) The concentration-dependent binding frequency of SOS_{FL} to a Ras bilayer.

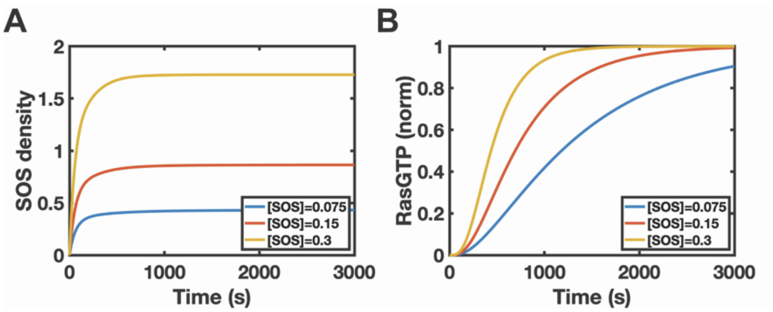

Figure S6. SOS concentration adjustment in simulation. [SOS_solution] = 0.15 works the best.

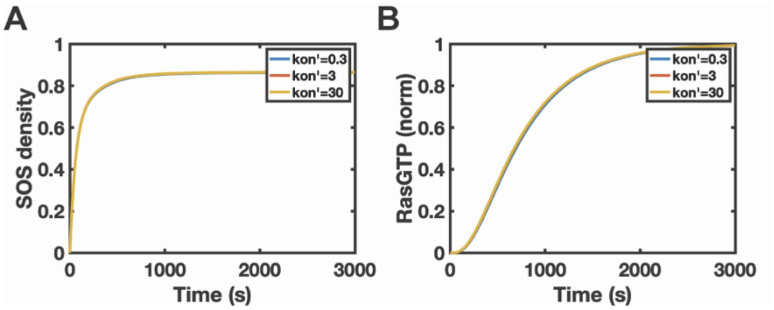

Figure S7. $k_{ras\_binding}$ estimation. Variation in kon' (or $k_{ras\_binding}$=kon'/[Ras_total]) does not have significant effects on SOS recruitment and Ras activation.

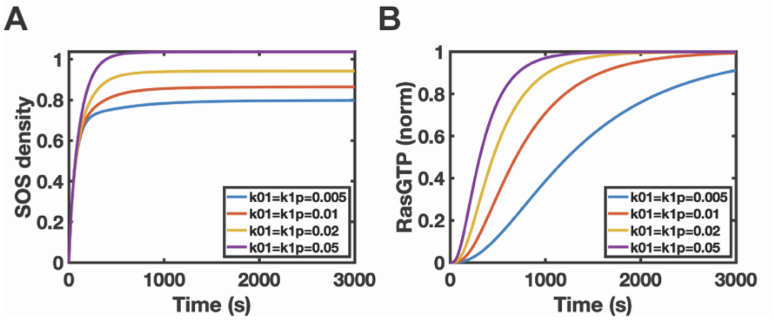

Figure S8. k_{01} and k_{1p} estimation. Various rates were tested, and k_{01}=k_{1p}=0.01 plot aligns with experimental measurement (Figure 3) the best.

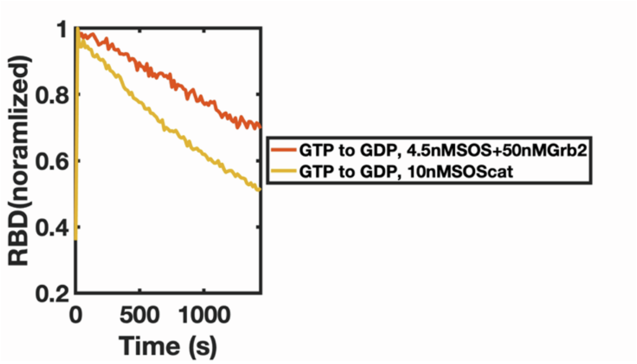

Figure S9. RasGTP to RasGDP nucleotide exchange reaction. First of all, it is not the natural reaction for SOS and the catalytic rate of SOS does exhibit nucleotide effects when swapping in GDP instead of GTP (9). Secondly, the SOS_FL autoinhibition release kinetics and dissociation rate once activated are quite slow, suggesting the reaction is not likely to exhibit significant non-equilibrium hysteresis effects even if it could be run symmetrically in reverse.

Reference

1. Cho Nathan, H., C. Cheveralls Keith, A.-D. Brunner, K. Kim, C. Michaelis André, P. Raghavan, H. Kobayashi, L. Savy, Y. Li Jason, H. Canaj, Y. S. Kim James, M. Stewart Edna, C. Gnann, F. McCarthy, P. Cabrera Joana, M. Brunetti Rachel, B. Chhun Bryant, G. Dingle, Y. Hein Marco, B. Huang, B. Mehta Shalin, S. Weissman Jonathan, R. Gómez-Sjöberg, N. Itzhak Daniel, A. Royer Loïc, M. Mann, and D. Leonetti Manuel. OpenCell: Endogenous tagging for the cartography of human cellular organization. *Science*. 375(6585):eabi6983, doi: 10.1126/science.abi6983
2. Seeliger, M. A., M. Young, M. N. Henderson, P. Pellicena, D. S. King, A. M. Falick, and J. Kuriyan. 2005. High yield bacterial expression of active c-Abl and c-Src tyrosine kinases. *Protein Science*. 14(12):3135-3139, doi: https://doi.org/10.1110/ps.051750905
3. Huang, W. Y. C., S. Alvarez, Y. Kondo, Y. K. Lee, J. K. Chung, H. Y. M. Lam, K. H. Biswas, J. Kuriyan, and J. T. Groves. 2019. A molecular assembly phase transition and kinetic proofreading modulate Ras activation by SOS. *Science*. 363(6431):1098-1103, doi: 10.1126/science.aau5721
4. Gureasko, J., W. J. Galush, S. Boykevisch, H. Sondermann, D. Bar-Sagi, J. T. Groves, and J. Kuriyan. 2008. Membrane-dependent signal integration by the Ras activator Son of sevenless. *Nature Structural & Molecular Biology*. 15(5):452-461, doi: 10.1038/nsmb.1418
5. Lin, C.-W., L. M. Nocka, B. L. Stinger, J. B. DeGrandchamp, L. J. N. Lew, S. Alvarez, H. T. Phan, Y. Kondo, J. Kuriyan, and J. T. Groves. 2022. A two-component protein condensate of the EGFR cytoplasmic tail and Grb2 regulates Ras activation by SOS at the membrane. *Proceedings of the National Academy of Sciences*. 119(19):e2122531119, doi: 10.1073/pnas.2122531119
6. Chung, J. K., Y. K. Lee, H. Y. M. Lam, and J. T. Groves. 2016. Covalent Ras Dimerization on Membrane Surfaces through Photosensitized Oxidation. *Journal of the American Chemical Society*. 138(6):1800-1803, doi: 10.1021/jacs.5b12648
7. Sondermann, H., S. M. Soisson, S. Boykevisch, S. S. Yang, D. Bar-Sagi, and J. Kuriyan. 2004. Structural analysis of autoinhibition in the Ras activator Son of sevenless. *Cell*. 119(3):393-405, doi: 10.1016/j.cell.2004.10.005
8. Vo, U., N. Vajpai, L. Flavell, R. Bobby, A. L. Breeze, K. J. Embrey, and A. P. Golovanov. 2016. Monitoring Ras Interactions with the Nucleotide Exchange Factor Son of Sevenless (Sos) Using Site-specific NMR Reporter Signals and Intrinsic Fluorescence*. *Journal of Biological Chemistry*. 291(4):1703-1718, doi: https://doi.org/10.1074/jbc.M115.691238
9. Iversen, L., H.-L. Tu, W.-C. Lin, M. Christensen Sune, M. Abel Steven, J. Iwig, H.-J. Wu, J. Gureasko, C. Rhodes, S. Petit Rebecca, D. Hansen Scott, P. Thill, C.-H. Yu, D. Stamou, K. Chakraborty Arup, J. Kuriyan, and T. Groves Jay. 2014. Ras activation by SOS: Allosteric regulation by altered fluctuation dynamics. *Science*. 345(6192):50-54, doi: 10.1126/science.1250373
