# Supplementary material for: Positive feedback in Ras activation by full-length SOS arises from autoinhibition release mechanism
Source: Biophys J. 2024 Jul 16;123(19):3295–303. doi: 10.1016/j.bpj.2024.07.014 (PMC11480760; doi:10.1016/j.bpj.2024.07.014)
Supplement: Document S2. Article plus supporting material [file mmc2.pdf]

Biophysical Journal
Article

Biophysical Society

Positive feedback in Ras activation by full-length SOS arises from autoinhibition release mechanism

He Ren,[1] Albert A. Lee,[2] L. J. Nugent Lew,[1] Joseph B. DeGrandchamp,[1] and Jay T. Groves[1,3,*]
[1]Department of Chemistry, University of California Berkeley, Berkeley, California; [2]Department of Molecular and Cell Biology, University of California Berkeley, Berkeley, California; and [3]Division of Molecular Biophysics and Integrated Bioimaging, Lawrence Berkeley National Laboratory, Berkeley, California

ABSTRACT Signaling through the Ras-MAPK pathway can exhibit switch-like activation, which has been attributed to the underlying positive feedback and bimodality in the activation of RasGDP to RasGTP by SOS. SOS contains both catalytic and allosteric Ras binding sites, and a common assumption is that allosteric activation selectively by RasGTP provides the mechanism of positive feedback. However, recent single-molecule studies have revealed that SOS catalytic rates are independent of the nucleotide state of Ras in the allosteric binding site, raising doubt about this as a positive feedback mechanism. Here, we perform detailed kinetic analyses of receptor-mediated recruitment of full-length SOS to the membrane while simultaneously monitoring its catalytic activation of Ras. These results, along with kinetic modeling, expose the autoinhibition release step in SOS, rather than either recruitment or allosteric activation, as the underlying mechanism giving rise to positive feedback in Ras activation.

SIGNIFICANCE Positive feedback is a prominent feature in the activation of Ras by SOS, and Ras binding to an allosteric site on SOS has long been considered a mechanism of this positive feedback. However, more recent experimental investigations of SOS allosteric activation have failed to reveal such effects. Detailed kinetic analyses of the full-length SOS protein now reveal its unique autoinhibition release process to be the primary source of positive feedback.

INTRODUCTION

Ras is a membrane-bound small GTPase that cycles between a GTP-bound on-state and a GDP-bound off-state (1). In the GTP-bound state, Ras can recruit downstream effector proteins to the membrane, leading to activation of the MAPK and PI3K pathways, which ultimately leads to cell growth and proliferation (2). Under nonactivating conditions, Ras resides in the GDP-bound state. Ras activation to the GTP-bound state is controlled by guanine nucleotide exchange factors (GEFs) that catalyze exchange of Ras-bound nucleotide with free nucleotide from the cytosol, which is primarily GTP (3). In the cellular setting, bimodality, or switch-like activity, in the Ras-MAPK signaling pathway has been shown to be important in lymphocytes (4) and contributes to the sharp boundary that discriminates positive and negative selection of thymocytes (5). Ras misregulation is a common cause of cancers, including lung cancer, colorectal cancer, and pancreatic cancer (6–8).

Son of Sevenless (SOS) is a key RasGEF, which operates downstream of both the growth factor receptor and T cell receptor pathways. The amine terminus of SOS consists of three regulatory domains: a histone fold (HF) domain (9), a Dbl homology (DH) domain, and a Pleckstrin homology (PH) domain (10). The REM-Cdc25 catalytic core sits in the middle (11) and is followed by a carboxy-terminal, structurally disordered proline-rich (PR) tail (12). Upon receptor activation, SOS is recruited via Grb2 to the cytosolic tail of epidermal growth factor receptor (EGFR) or, in the case of T cell receptor signaling, to Linker for activation of T cells (LAT). In both cases, SH3 domains on the adaptor protein Grb2 bind to the PR domain of SOS, while the Grb2 SH2 domain binds to phospho-tyrosine sites on the active receptor or scaffold (13,14). Once on the membrane, SOS can initiate the nucleotide exchange reaction after a multistep autoinhibition release process (Fig. 1) (15).

Submitted March 24, 2024, and accepted for publication July 12, 2024.

*Correspondence: jtgroves@lbl.gov

Albert A. Lee's present address is Department of Biochemistry, Stanford University, Stanford, California

Joseph B. DeGrandchamp's present address is Institute for Digital Molecular Analytics and Science, Nanyang Technological University, Singapore

Editor: William Hancock.

https://doi.org/10.1016/j.bpj.2024.07.014

© 2024 The Author(s). Published by Elsevier Inc. on behalf of Biophysical Society.
This is an open access article under the CC BY license (http://creativecommons.org/licenses/by/4.0/).

An unusual feature of SOS, compared with other RasGEFs, is that it has two Ras binding sites in its REM-Cdc25 catalytic core (16). There is one catalytic site at the Cdc25 domain and one noncatalytic allosteric site positioned between the REM and Cdc25 domains (11,15). Structural studies have shown that allosteric Ras binding is essential for SOS catalytic activity (17,18). In addition, both solution and liposome nucleotide exchange assays using the catalytic core of SOS have shown positive feedback in the activation of Ras by SOS. The higher binding affinity for RasGTP relative to RasGDP at the SOS allosteric site as well as a presumption of nucleotide-specific enhancement of catalytic activity have generally been accepted as the mechanisms of positive feedback (4,18–21). However, these observations were made with truncated SOS constructs lacking autoinhibition and membrane recruitment through accessory domains. Recent studies have started to provide detailed characterizations of the full-length SOS (SOS_{FL}) molecule (22,23).

Single-molecule membrane microarray studies of SOS have revealed several facets of its functional mechanism that were not evident in earlier work. First, SOS is highly processive and can activate hundreds of Ras molecules in a single membrane binding event (22,24,25). Second, the catalytic rate of Ras activation by an individual SOS molecule is independent of the nucleotide state of Ras in the allosteric binding site (24). Third, SOS_{FL} exhibits an autoinhibition release process on the membrane that introduces a long delay (tens of seconds) between initial recruitment of SOS to the membrane and onset of its processive catalytic activity. Analyses of the delay time indicates that this slow activation process consists of multiple steps occurring far from equilibrium (23). This slow autoinhibition release feature of SOS enables a novel type of kinetic proofreading regulation of SOS activity, which is under the control of a protein condensation phase transition of the recruiting EGFR or LAT molecules (23,26,27). Collectively, this body of work experimentally demonstrates that SOS_{FL} lacks the classically presumed positive feedback in allosteric enhancement of its catalytic rate. SOS_{FL} does, however, exhibit marked positive feedback in Ras activation (28), raising questions of what the actual underlying mechanism is.

In this study, we performed detailed kinetic analyses of receptor-mediated recruitment and activity of SOS_{FL} in a supported membrane platform. Using total internal reflection fluorescence (TIRF) microscopy, we monitor the recruitment (via Grb2) of SOS_{FL} to phosphorylated LAT scaffold molecules on the membrane while simultaneously tracking the Ras activity state using a modified Ras binding domain from Raf-1 (residues 56–131, K65E; referred to as RBD hereafter). This RBD construct exhibits substantially faster binding kinetics compared with wild-type and provides faithful real-time readout of RasGTP densities on the membrane (23). The results confirm that receptor-mediated recruitment of SOS_{FL} is independent of the Ras-nucleotide state. Furthermore, we obtain quantitative measurements of the effective catalytic rate of a defined ensemble of membrane-recruited SOS_{FL} molecules as a function of RasGTP density, which confirms the existence of positive feedback at the ensemble level that is not observed in the individual molecules (24). Finally, we developed a minimal kinetic model that incorporates details of the SOS_{FL} autoinhibition release mechanism, which successfully recapitulates the experimental observations. From these results, we conclude that the observed positive feedback in Ras activation by receptor-recruited SOS_{FL} is an ensemble effect stemming from RasGTP-mediated enhancement in the rate of autoinhibition release. Recently, several small-molecule inhibitors that target the SOS:Ras interaction have emerged as candidates for pan-KRas cancer therapy (29,30). Developing a comprehensive understanding of the mechanism underlying Ras activation by SOS may be useful for understanding the behavior SOS:Ras targeting drugs.

RESULTS

The receptor-mediated recruitment of SOS_{FL} is not sensitive to Ras nucleotide state

Although the catalytic activity of SOS does not exhibit Ras-nucleotide state sensitivity at the single-molecule level (24), enhanced membrane recruitment provides an alternative mechanism for positive feedback. The SOS catalytic core (SOS_{CAT}) and SOS_{HDPC} constructs, both of which lack the C-terminal PR domain responsible for receptor-mediated membrane recruitment (see SOS domain structure in Fig. 1), are primarily recruited to the membrane through binding Ras and exhibit preference for RasGTP. Recruitment of enzymes that act on membrane substrates to the membrane via their own product is a direct form of positive feedback (31,32). Increased recruitment to the RasGTP-containing membrane is a likely source for the observed positive feedback in SOS_{CAT} and SOS_{HDPC}. The native SOS_{FL}, however, is robustly autoinhibited—including with respect to Ras binding (17,18,22,33)—and is primarily recruited to the membrane via Grb2-mediated binding to phosphorylated receptor or scaffold proteins on the membrane (LAT in the experiments described here, as shown in Fig. 1). Thus, the native autoinhibition in SOS_{FL} is expected to prevent the recruitment-based positive feedback observed in truncated SOS constructs. To test this hypothesis, we perform detailed kinetic analysis of SOS recruitment in a supported bilayer configuration (22–25,34).

Purified Ras was tethered to the supported lipid bilayer through cysteine-maleimide chemistry at a density ~2000 molecule per μm^2, following established protocols (24). Ras was preincubated with unlabeled SOS_{CAT} to control the initial nucleotide state. Residual SOS_{CAT} was thoroughly washed away before initiating the recruitment assay. To

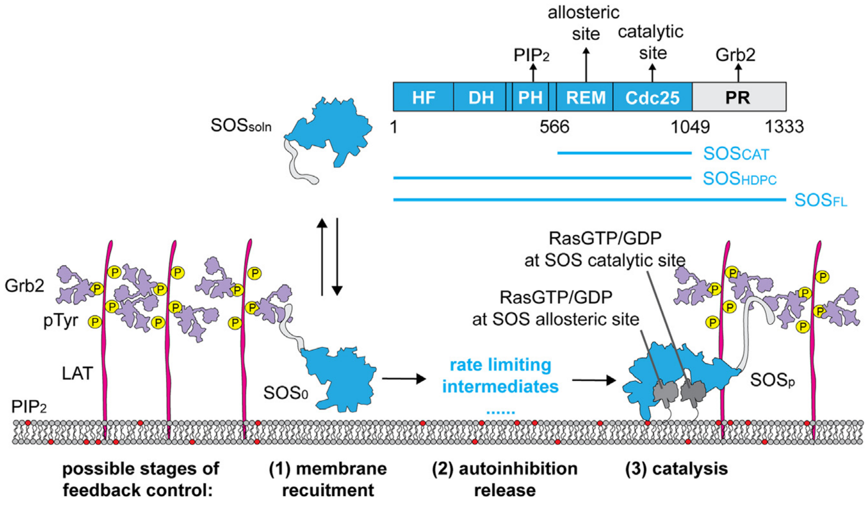

FIGURE 1 The domain organization and activation process of SOS. SOS_{FL} stays in an autoinhibited conformation in solution (SOS_{soln}). After T cell receptor stimulation and LAT phosphorylation, SOS_{FL} activation follows initial membrane recruitment in an autoinhibited conformation (SOS_0), slow autoinhibition release on the membrane, and processive Ras nucleotide exchange after activation (SOS_p). These three steps of activation provide potential mechanisms for feedback control in SOS.

ensure similar densities of proteins incubated on the membrane, flow chambers for RasGDP and RasGTP were prepared side by side. SOS constructs were labeled with Alexa Fluor 555 or Alexa Fluor 647 and the recruitment of SOS to the membrane was quantified using single-molecule counting via TIRF microscopy. Excess nucleotide was included in each flow chamber (GDP in the RasGDP chamber, and vice-versa for the RasGTP chamber) so that the nucleotide state of Ras would not change over the course of the measurement. In the SOS_{FL} recruitment experiment, the cytosolic domain of scaffold protein LAT (with N-terminal His10 tag (35)) was linked to the bilayer through nickel-histidine interaction to achieve experimental densities of ~300–1000 molecules per μm^2. LAT was maintained in a fully phosphorylated state by the promiscuous Src family kinase, Hck (with His6 tag), which was also on the bilayer (26). In solution, Grb2 was included in the system at 20 nM to enable recruitment of SOS_{FL} to the membrane through a LAT·Grb2·SOS_{FL} complex, which we refer to as receptor-mediated recruitment. The experiments were performed with physiological concentrations of LAT (in condensates) (36), Ras (as reported in Ras nanoclusters) (37), Grb2, and SOS (38). In reconstitution, fully percolated condensates that span the entire sample can be created (23,27), but the experiments described here were performed toward the dispersed end of this spectrum. Since SOS_{FL} alone can cross-link multiple Grb2·LAT, there is still microscopic LAT clustering and multivalent SOS engagement in these experiments—even while macroscopically extended condensates are not seen.

Measurements of Ras-mediated recruitment of Alexa647-labeled SOS_{CAT} and Alexa647-labeled SOS_{HDPC} to RasGTP or RasGDP functionalized membranes confirm a strong preference by SOS_{CAT} for RasGTP and a mild preference by SOS_{HDPC} (Fig. 2, *A–C*). Experiments at higher SOS_{HDPC} concentrations are shown in Fig. S1 *A*. Some of this observed Ras-nucleotide preference likely stems from the binding affinity differences between SOS-RasGTP and SOS-RasGDP at equilibrium (18,19). However, single-molecule SOS studies have revealed that SOS has several internal states, whereby a subset of membrane recruited molecules become stably associated with the membrane and enter a processive, catalytically active state while others exhibit only transient membrane interactions (22,34). Thus, the ratio between processive and transient states of SOS will also affect equilibrium density differentially between different SOS constructs (34). The discrepancy in the extent of Ras-nucleotide preference between SOS_{CAT} and SOS_{HDPC} may also stem from the accessibility of Ras binding site, as the regulatory domains keep SOS_{HDPC} in a more autoinhibited conformation than SOS_{CAT} (18). In addition, PI(4,5)P_2 and PH domain interaction (33,39) may enhance SOS_{HDPC} membrane association in a Ras-independent manner.

In contrast to SOS_{CAT} and SOS_{HDPC}, SOS_{FL} clearly exhibits Ras nucleotide-independent recruitment to the membrane (Fig. 2, *D* and *E*). Another experiment at a higher SOS_{FL} concentration is shown in Fig. S1 *B*. SOS_{FL} is primarily recruited via Grb2 to phosphorylated LAT on the membrane and exhibits minimal membrane binding in the absence of Grb2 (Fig. 2 *F*). In addition, there was no measurable effect in the initial SOS recruitment kinetics due to Ras density (Fig. S2). From the SOS_{FL} membrane recruitment data in Fig. 2 *E*, it is evident that both the rise time (reflective of the kinetic on-rate for membrane recruitment) and the plateau density (reflective of equilibrium) are independent of Ras nucleotide state. This also directly shows that the dissociation rate, or kinetic off-rate, of SOS_{FL} is insensitive to the Ras nucleotide state as well. Therefore, even though the SOS_{FL} allosteric site has a higher binding affinity for RasGTP, this experimental evidence indicates that such preference does not contribute significantly to SOS_{FL} membrane recruitment. Thus, SOS_{FL} does not exhibit positive feedback in receptor-mediated membrane recruitment.

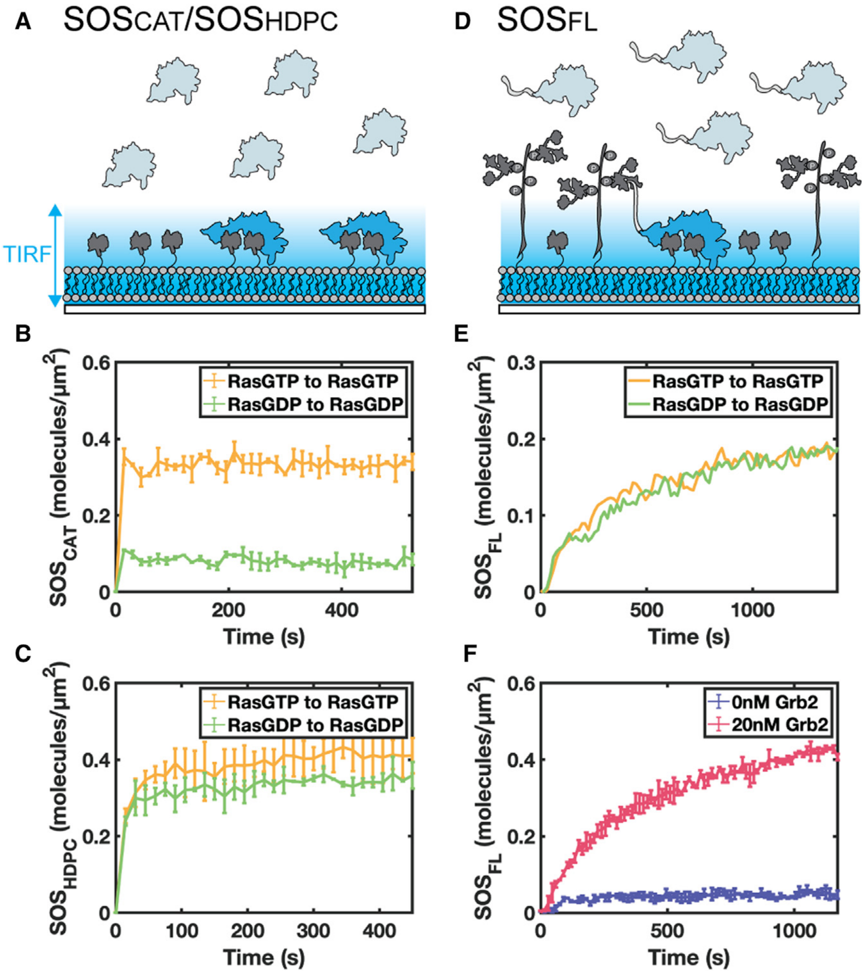

FIGURE 2 The receptor-mediated recruitment of SOS_{FL} is not sensitive to Ras nucleotide state. (*A*) Schematic of SOS_{CAT} or SOS_{HDPC} recruitment to Ras functionalized supported membrane. TIRF microscopy allows the quantification of SOS membrane recruitment under different membrane composition. Ras nucleotide-dependent membrane recruitment of (*B*) SOS_{CAT} (n = 2) and (*C*) SOS_{HDPC} (n = 2). (*B*) and (*C*) do not include LAT and Grb2. (*D*) Schematic of SOS_{FL} recruitment to pLAT and Ras functionalized supported membrane via Grb2. Both (*E*) and (*F*) include LAT and Grb2. (*E*) Ras nucleotide-independent membrane recruitment of SOS_{FL} (n = 1). (*F*) SOS_{FL} recruitment depends on Grb2 concentration (n = 2). Experiments on each plot were conducted on the same day to prevent significant fluctuations in membrane protein density. The error bars represent the standard deviation of the runs.

Ensemble catalytic activity of SOS_{FL} exhibits RasGTP-driven positive feedback

Ras activation studies by SOS_{FL} were performed similarly as the recruitment assays discussed above, except that we simultaneously measured SOS_{FL}-catalyzed Ras nucleotide exchange from RasGDP to RasGTP (Fig. 3 *A*). RasGTP density was measured by the membrane recruitment of a fluorescently labeled RBD sensor, adapted from Raf with K65E point mutation to ensure fast binding and unbinding kinetics to Ras (23). The RBD sensor provides a real-time readout of RasGTP levels in the system, and was calibrated to directly reflect RasGTP density as measured by fluorescence correlation spectroscopy in Fig. S3. When the RBD sensor intensity reaches a plateau, the readout indicates the total Ras level, as all the RasGDP has been converted to RasGTP. This defines the RasGTP/total Ras ratio. RasGTP levels and SOS_{FL} membrane density (Fig. 3 *B*) were simultaneously measured. Example time-lapse images for RBD and SOS_{FL} membrane recruitment are shown in Fig. 3 *C*.

Ras densities are significantly higher than SOS_{FL} densities in these experiments and SOS_{FL} is working under substrate saturating conditions (24). Under these conditions, the overall rate of RasGTP production is first order with SOS density and proportional to the overall SOS turnover rate by the ratio of RasGDP/Ras_{total}. This proportionality results from the fact that, even when the catalytic site of SOS binds RasGTP, it still spends an entire enzymatic cycle to exchange GTP for GTP, with no net contribution to the overall RasGTP production rate. The following rate equation was used to calculate the per-molecule catalytic rate of SOS_{FL}:

$$\frac{\mathrm{dRasGTP}}{\mathrm{dt}} = k_{obs} \times \sigma[\mathrm{SOS}] \times \sigma[\mathrm{RasGDP}] / \sigma[\mathrm{Ras_{total}}] \tag{1}$$

where k_{obs} is the ensemble average catalytic turnover per SOS_{FL} molecule, σ denotes surface density. The increasing apparent per-molecule catalytic rate (Fig. 3 *D*) shows that SOS_{FL} exhibits RasGTP-dependent positive feedback, but evidently not by the same mechanism as SOS_{CAT} and SOS_{HDPC}.

With membrane recruitment and molecular catalytic activity already eliminated as possible mechanisms for SOS_{FL}

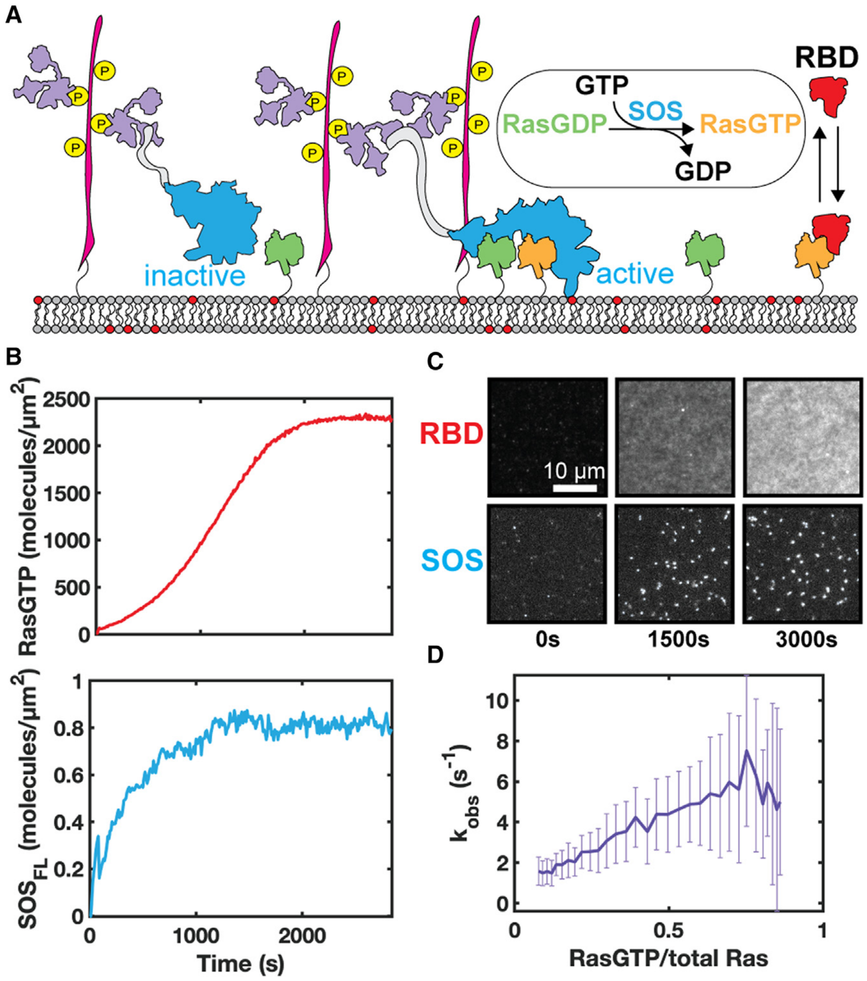

FIGURE 3 SOS_{FL} autoinhibition release depends on RasGTP levels. (*A*) Schematic of the activity assay for determining the fraction of active SOS on the membrane. Recruited SOS_{FL} is either in the process of autoinhibition release or in the active conformation. RBD sensor acts as a real-time RasGTP level readout. (*B*) Ras activation inferred from RBD recruitment and SOS membrane density were measured simultaneously. (*C*) Example time-lapse images for RBD and SOS recruitment. (*D*) Ensemble average catalytic rate (k_{obs}) for all SOS_{FL} recruited to the membrane at different RasGTP level. For the k_{obs} calculation, the RasGTP level and SOS_{FL} membrane density were averaged every five time points, and the standard deviation was calculated and propagated as the error, represented by the error bar.

positive feedback, the only remaining mechanistic possibility is the autoinhibition release process. SOS_{FL} is recruited to the membrane in an autoinhibited state (here referred to as SOS_0 for kinetic modeling purposes). After initial membrane recruitment, the simplest model for the autoinhibition release process involves one dominant rate-limiting kinetic intermediate (SOS_1) before entering its active state (SOS_p), during which SOS begins to processively catalyze nucleotide exchange in Ras (23). If RasGTP levels on the membrane alter the kinetics of SOS progression to its activated state, then this provides an alternative mechanism for RasGTP-driven positive feedback. We examine this possibility quantitatively with kinetic modeling.

The proposed kinetic scheme is depicted in Fig. 4 *A*. SOS_{FL} recruitment and progression to its kinetic intermediate (SOS_1) state are represented as Ras density independent. SOS_1 then binds RasGTP or RasGDP at the allosteric binding site and the final transition to SOS_p has different rates depending on the nucleotide state of allosterically bound Ras. This is the presumed sequence since $PI(4,5)P_2$ binding releases the DH-PH domain and exposes the Ras allosteric binding pocket (33,40). All membrane-bound SOS molecules may dissociate back into the solution with rates that depend on their activity state. The relaxation time back to the autoinhibited state once a SOS molecule has desorbed is short compared with the recycling time of SOS_{FL} back to the membrane. Therefore, all the newly bound SOS molecules start in an autoinhibited conformation. The choice to model SOS activation as a one-way process builds upon a previous study of ours (41), in which we confirmed that allowing reversibility in the activation intermediate steps does not qualitatively change SOS activation behavior. In addition, under physiological conditions, the SOS activation process occurs out of equilibrium with a net flux toward activation. The rate equations for each membrane-bound species are listed in the supporting information. The temporal evolution of the system was numerically solved as differential equations in MATLAB.

Based on the proposed model, there are three potential steps giving rise to positive feedback: these include Ras binding to the SOS allosteric site (k_5 and k_6), Ras-bound SOS entering the processively active state (k_7 and k_8), and Ras-bound SOS desorbing from the membrane (k_9, k_{10},

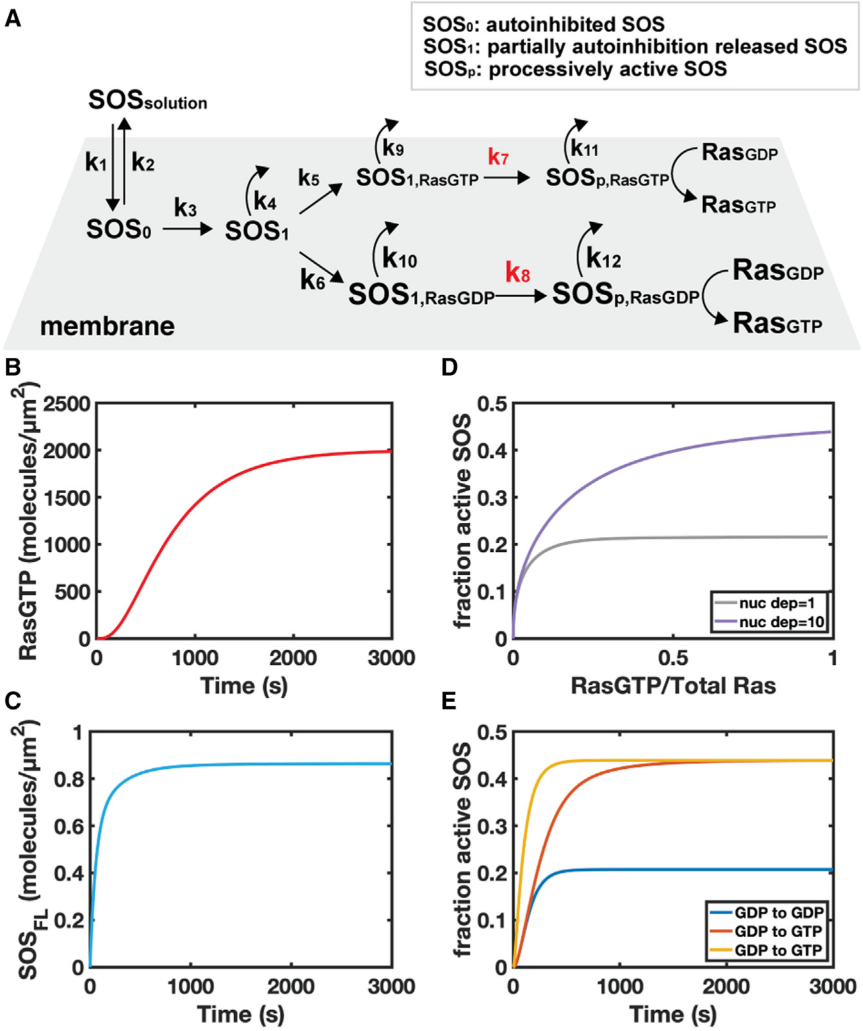

FIGURE 4 Kinetic simulations show accelerated autoinhibition release as a mechanism for positive feedback in SOS activation. (*A*) Schematic for the kinetic model. (*B*) Plot of RasGTP level over time in RasGDP to RasGTP nucleotide exchange reaction from simulation. (*C*) Plot of SOS membrane recruitment over time, obtained simultaneously with Ras activity. (*D*) Plot of fraction of active SOS over time, obtained from the ratio between processively active SOS and total SOS on the membrane. (*E*) Plots of Ras nucleotide-dependent activation of SOS.

k_{11}, and k_{12}). Since we inferred from the SOS_{FL} receptor-mediated recruitment assay that the membrane dissociation rate of SOS_{FL} is insensitive to the Ras nucleotide state (Fig. 2 *E*), we focused on the first two mechanisms in the simulation.

Ras binding to the SOS allosteric site at equilibrium has been the subject of numerous studies investigating the details of positive feedback in Ras activation by SOS (18–20). However, the direct measurement of the kinetics of this binding process (k_5 and k_6) is lacking. We utilized the kinetics of SOS_{CAT} membrane recruitment obtained in the previous section to estimate the range of possible rates for SOS_1 binding to Ras on the membrane (details shown in the supporting material). On both RasGTP and RasGDP bilayers, SOS_{CAT} reached a steady state in less than 15 s (Fig. 2 *B*). The binding rates to RasGTP and RasGDP were adjusted proportionally based on the difference in binding affinity of SOS_{CAT} (18). Although membrane adsorption kinetics are not directly transferable to collision rates on the membrane, this estimation reveals that the Ras binding step is a rapid process, significantly shorter than the time scale required for SOS activation (23). Consequently, we concluded that Ras binding is not a rate-limiting step in SOS activation. The transition rates of Ras-bound SOS into the processively active state (k_7 and k_8) have also not been experimentally measured before. Nevertheless, single-molecule measurements (23) indicate that the time scale for membrane recruited SOS_{FL} to reach the active state is on the order of tens of seconds.

Other kinetic parameters were derived from previous experimental measurements and adjusted to align with observed experimental data. The rate of solution SOS binding to the membrane (k_1) was estimated using the early time points from our recruitment traces (Fig. S5). The rate of membrane dissociation of inactive SOS molecules (SOS_0, SOS_1, and $SOS_{1,RasGXP}$) were all treated to be equal to a rate ($k_{off\_transient}$) that was measured from a previous experimental study (23). While improvements in these parameters by considering additional membrane interactions would

likely lead to more accurate simulations, we have opted not to extend the simulation details beyond the level of available experimental input for this model. The membrane dissociation rates of active SOS_{FL} molecules ($SOS_{p,\ RasGXP}$), $k_{off\_processive}$, were estimated from single-molecule activation profiles (34).

After adjusting the parameters (details in the supporting material), we introduced a nucleotide preference to k_7 and k_8 ($k_7 = 10\times\ k^8$). The simulation results confirm that accelerated autoinhibition release can indeed serve as a mechanism for positive feedback in activation of Ras by SOS. Firstly, the nucleotide exchange reactions followed a time scale similar to that observed in experiments, and the overall SOS recruitment reached a similar level (Fig. 4, *B* and *C*). Secondly, the fraction of active SOS, which is directly proportional to the apparent average catalytic rate per SOS molecule (see supporting material for details), increased with higher RasGTP levels. RasGTP-enhanced SOS activation is not observed when the nucleotide preference is set to 1 (Fig. 4 *D*). Moreover, the simulation allowed us to calculate the fraction of active SOS during homonucleotide exchange (RasGTP to RasGTP and RasGDP to RasGDP; Fig. 4 *E*), which could not be experimentally measured. The simulation results revealed that the system reached a steady state with a higher fraction of active SOS when cycling RasGTP to RasGTP since all SOS activations occurred through the accelerated pathway. In contrast, in the RasGDP homonucleotide exchange condition (RasGDP to RasGDP), the fraction of activated SOS remained low throughout the simulation. A shift from a low fraction of active SOS to a high fraction was observed in the RasGDP to RasGTP simulation as more RasGTP was produced from the nucleotide exchange reaction, resembling the positive feedback seen in the Ras activation by SOS_{FL} experiments.

DISCUSSION AND CONCLUSION

In this study, we have resolved a distinct mechanistic source of positive feedback in the native SOS_{FL} protein that contrasts behavior of its truncated forms. SOS has long been known to play an important role in the bimodal behavior of signaling through Ras and the MAPK pathway (4,5). Although SOS has a preference toward RasGTP binding at the allosteric site (18), which would seem to be the most obvious source of positive feedback in its Ras activation kinetics, this preference becomes inconsequential in the native SOS_{FL} due to its autoinhibition. In addition, single-molecule experiments have demonstrated that SOS catalytic activity is independent of Ras-nucleotide state at the molecular level—ruling this out as a positive feedback mechanism. Here, we have shown, through both experiments and kinetic modeling, that it is the autoinhibition release process in SOS_{FL} that provides the dominant source of positive feedback.

A few other recent studies have also revisited the origin of the positive feedback in Ras activation by SOS. The studies by Vo et al. (19) and Liao et al. (20) focused on the interaction between Ras and SOS catalytic site and allosteric site, suggesting that RasGDP is a weaker binder than RasGTP toward the allosteric site, but a stronger binder toward the catalytic site. However, preferential binding of RasGDP to the catalytic site is not likely a driver of the positive feedback observed in the experiments described here, which were all performed at high Ras density where the catalytic nucleotide exchange step—not the RasGDP binding to the catalytic site—is rate limiting.

Another paper from our own group (34) has examined reconstituted bimodality in a competitive Ras activation-deactivation assay. In that work, processivity of SOS was further identified as a key driver of bimodality in the competitive reaction. Taken together, all these recent studies and the present work reveal a multifaceted mechanism of positive feedback in the activation of Ras by SOS. Although simple effects of allosteric activation by preferential binding of RasGTP to the allosteric binding site clearly leads to positive feedback in truncated SOS variants, the SOS_{FL} protein is much more complex and relies heavily on autoinhibition release and processivity to achieve its macroscopic behavior.

SUPPORTING MATERIAL

Supporting material can be found online at https://doi.org/10.1016/j.bpj.2024.07.014.

AUTHOR CONTRIBUTIONS

H.R., A.A.L., and J.T.G. designed the study. H.R., A.A.L., L.J.N.L., and J.B.D. prepared reagent. H.R. performed experimental data collection. H.R. performed kinetic modeling. H.R. analyzed data. H.R. and J.T.G. wrote the manuscript. All authors edited and approved the manuscript.

ACKNOWLEDGMENTS

We thank the members of the Groves Lab for helpful discussion and critical feedback on the manuscript. We thank John Kuriyan for providing the plasmids for SOS derivatives. This work was supported by the Novo Nordisk Foundation Challenge Program under the Center for Geometrically Engineered Cellular Systems. Additional support was provided by the NIH NIAID grant P01 A1091580.

DECLARATION OF INTERESTS

All authors declare they have no competing interests.

REFERENCES

1. Bourne, H. R., D. A. Sanders, and F. McCormick. 1990. The GTPase superfamily: a conserved switch for diverse cell functions. *Nature*. 348:125–132. https://doi.org/10.1038/348125a0.

2. Simanshu, D. K., D. V. Nissley, and F. McCormick. 2017. RAS Proteins and Their Regulators in Human Disease. *Cell.* 170:17–33. https://doi.org/10.1016/j.cell.2017.06.009.

3. Bos, J. L., H. Rehmann, and A. Wittinghofer. 2007. GEFs and GAPs: Critical Elements in the Control of Small G Proteins. *Cell.* 129:865–877. https://doi.org/10.1016/j.cell.2007.05.018.

4. Das, J., M. Ho, …, J. P. Roose. 2009. Digital Signaling and Hysteresis Characterize Ras Activation in Lymphoid Cells. *Cell.* 136:337–351. https://doi.org/10.1016/j.cell.2008.11.051.

5. Prasad, A., J. Zikherman, …, A. K. Chakraborty. 2009. Origin of the sharp boundary that discriminates positive and negative selection of thymocytes. *Proc. Natl. Acad. Sci. USA.* 106:528–533. https://doi.org/10.1073/pnas.0805981105.

6. Prior, I. A., P. D. Lewis, and C. Mattos. 2012. A Comprehensive Survey of Ras Mutations in Cancer. *Cancer Res.* 72:2457–2467. https://doi.org/10.1158/0008-5472.CAN-11-2612.

7. Hobbs, G. A., C. J. Der, and K. L. Rossman. 2016. RAS isoforms and mutations in cancer at a glance. *J. Cell Sci.* 129:1287–1292. https://doi.org/10.1242/jcs.182873.

8. Moore, A. R., S. C. Rosenberg, …, S. Malek. 2020. RAS-targeted therapies: is the undruggable drugged? *Nat. Rev. Drug Discov.* 19:533–552. https://doi.org/10.1038/s41573-020-0068-6.

9. Sondermann, H., B. Nagar, …, J. Kuriyan. 2005. Computational docking and solution x-ray scattering predict a membrane-interacting role for the histone domain of the Ras activator son of sevenless. *Proc. Natl. Acad. Sci. USA.* 102:16632–16637. https://doi.org/10.1073/pnas.0508315102.

10. Soisson, S. M., A. S. Nimnual, …, J. Kuriyan. 1998. Crystal Structure of the Dbl and Pleckstrin Homology Domains from the Human Son of Sevenless Protein. *Cell.* 95:259–268. https://doi.org/10.1016/S0092-8674(00)81756-0.

11. Margarit, S. M., H. Sondermann, …, J. Kuriyan. 2003. Structural Evidence for Feedback Activation by RasGTP of the Ras-Specific Nucleotide Exchange Factor SOS. *Cell.* 112:685–695. https://doi.org/10.1016/S0092-8674(03)00149-1.

12. McDonald, C. B., V. Bhat, …, A. Farooq. 2013. Structural landscape of the proline-rich domain of Sos1 nucleotide exchange factor. *Biophys. Chem.* 175–176:54–62. https://doi.org/10.1016/j.bpc.2013.02.008.

13. Lowenstein, E. J., R. J. Daly, …, J. Schlessinger. 1992. The SH2 and SH3 domain-containing protein GRB2 links receptor tyrosine kinases to ras signaling. *Cell.* 70:431–442. https://doi.org/10.1016/0092-8674(92)90167-B.

14. Chardin, P., J. H. Camonis, …, D. Bar-Sagi. 1993. Human Sos1: a Guanine Nucleotide Exchange Factor for Ras that Binds to GRB2. *Science.* 260:1338–1343. https://doi.org/10.1126/science.8493579.

15. Bandaru, P., Y. Kondo, and J. Kuriyan. 2019. The Interdependent Activation of Son-of-Sevenless and Ras. *Cold Spring Harb. Perspect. Med.* 9:a031534. https://doi.org/10.1101/cshperspect.a031534.

16. Jun, J., I. Rubio, and J. Roose. 2013. Regulation of Ras Exchange Factors and Cellular Localization of Ras Activation by Lipid Messengers in T Cells. *Front. Immunol.* 4:239.

17. Freedman, T. S., H. Sondermann, …, J. Kuriyan. 2006. A Ras-induced conformational switch in the Ras activator Son of sevenless. *Proc. Natl. Acad. Sci. USA.* 103:16692–16697. https://doi.org/10.1073/pnas.0608127103.

18. Sondermann, H., S. M. Soisson, …, J. Kuriyan. 2004. Structural analysis of autoinhibition in the Ras activator Son of sevenless. *Cell.* 119:393–405. https://doi.org/10.1016/j.cell.2004.10.005.

19. Vo, U., N. Vajpai, …, A. P. Golovanov. 2016. Monitoring Ras Interactions with the Nucleotide Exchange Factor Son of Sevenless (Sos) Using Site-specific NMR Reporter Signals and Intrinsic Fluorescence. *J. Biol. Chem.* 291:1703–1718. https://doi.org/10.1074/jbc.M115.691238.

20. Liao, T.-J., H. Jang, …, R. Nussinov. 2018. Allosteric KRas4B Can Modulate SOS1 Fast and Slow Ras Activation Cycles. *Biophys. J.* 115:629–641. https://doi.org/10.1016/j.bpj.2018.07.016.

21. Hoang, H. M., H. G. Umutesi, and J. Heo. 2021. Allosteric autoactivation of SOS and its kinetic mechanism. *Small GTPases.* 12:44–59. https://doi.org/10.1080/21541248.2019.1601954.

22. Lee, Y. K., S. T. Low-Nam, …, J. T. Groves. 2017. Mechanism of SOS PR-domain autoinhibition revealed by single-molecule assays on native protein from lysate. *Nat. Commun.* 8:15061. https://doi.org/10.1038/ncomms15061.

23. Huang, W. Y. C., S. Alvarez, …, J. T. Groves. 2019. A molecular assembly phase transition and kinetic proofreading modulate Ras activation by SOS. *Science.* 363:1098–1103. https://doi.org/10.1126/science.aau5721.

24. Iversen, L., H.-L. Tu, …, T. Groves Jay. 2014. Molecular kinetics. Ras activation by SOS: allosteric regulation by altered fluctuation dynamics. *Science.* 345:50–54. https://doi.org/10.1126/science.1250373.

25. Christensen, S. M., H.-L. Tu, …, J. T. Groves. 2016. One-way membrane trafficking of SOS in receptor-triggered Ras activation. *Nat. Struct. Mol. Biol.* 23:838–846. https://doi.org/10.1038/nsmb.3275.

26. Huang, W. Y. C., Q. Yan, …, J. T. Groves. 2016. Phosphotyrosine-mediated LAT assembly on membranes drives kinetic bifurcation in recruitment dynamics of the Ras activator SOS. *Proc. Natl. Acad. Sci. USA.* 113:8218–8223. https://doi.org/10.1073/pnas.1602602113.

27. Lin, C.-W., L. M. Nocka, …, J. T. Groves. 2022. A two-component protein condensate of the EGFR cytoplasmic tail and Grb2 regulates Ras activation by SOS at the membrane. *Proc. Natl. Acad. Sci. USA.* 119:e2122531119. https://doi.org/10.1073/pnas.2122531119.

28. Boykevisch, S., C. Zhao, …, D. Bar-Sagi. 2006. Regulation of Ras Signaling Dynamics by Sos-Mediated Positive Feedback. *Curr. Biol.* 16:2173–2179. https://doi.org/10.1016/j.cub.2006.09.033.

29. Hillig, R. C., B. Sautier, …, B. Bader. 2019. Discovery of potent SOS1 inhibitors that block RAS activation via disruption of the RAS–SOS1 interaction. *Proc. Natl. Acad. Sci. USA.* 116:2551–2560. https://doi.org/10.1073/pnas.1812963116.

30. Hofmann, M. H., M. Gmachl, …, N. Kraut. 2021. BI-3406, a Potent and Selective SOS1–KRAS Interaction Inhibitor, Is Effective in KRAS-Driven Cancers through Combined MEK Inhibition. *Cancer Discov.* 11:142–157. https://doi.org/10.1158/2159-8290.CD-20-0142.

31. Hansen, S. D., W. Y. C. Huang, …, J. T. Groves. 2019. Stochastic geometry sensing and polarization in a lipid kinase-phosphatase competitive reaction. *Proc. Natl. Acad. Sci. USA.* 116:15013–15022. https://doi.org/10.1073/pnas.1901744116.

32. Lee, A. A., W. Y. C. Huang, …, J. T. Groves. 2021. Stochasticity and positive feedback enable enzyme kinetics at the membrane to sense reaction size. *Proc. Natl. Acad. Sci. USA.* 118:e2103626118. https://doi.org/10.1073/pnas.2103626118.

33. Gureasko, J., O. Kuchment, …, J. Kuriyan. 2010. Role of the histone domain in the autoinhibition and activation of the Ras activator Son of Sevenless. *Proc. Natl. Acad. Sci. USA.* 107:3430–3435. https://doi.org/10.1073/pnas.0913915107.

34. Lee, A. A., N. H. Kim, …, J. T. Groves. 2024. Bimodality in Ras signaling originates from processivity of the Ras activator SOS without deterministic bistability. *Sci. Adv.* 10:eadi0707. https://doi.org/10.1126/sciadv.adi0707.

35. Nye, J. A., and J. T. Groves. 2008. Kinetic Control of Histidine-Tagged Protein Surface Density on Supported Lipid Bilayers. *Langmuir.* 24:4145–4149. https://doi.org/10.1021/la703788h.

36. McAffee, D. B., M. K. O'Dair, …, J. T. Groves. 2022. Discrete LAT condensates encode antigen information from single pMHC:TCR binding events. *Nat. Commun.* 13:7446. https://doi.org/10.1038/s41467-022-35093-9.

37. Gurry, T., O. Kahramanoğulları, and R. G. Endres. 2009. Biophysical Mechanism for Ras-Nanocluster Formation and Signaling in Plasma Membrane. *PLoS One.* 4:e6148. https://doi.org/10.1371/journal.pone.0006148.

38. Shi, T., M. Niepel, …, H. S. Wiley. 2016. Conservation of protein abundance patterns reveals the regulatory architecture of the EGFR-MAPK pathway. *Sci. Signal.* 9:rs6. https://doi.org/10.1126/scisignal.aaf0891.

39. Kubiseski, T. J., Y. M. Chook, …, T. Pawson. 1997. High Affinity Binding of the Pleckstrin Homology Domain of mSos1 to Phosphatidylinositol (4,5)-Bisphosphate. *J. Biol. Chem.* 272:1799–1804. https://doi.org/10.1074/jbc.272.3.1799.

40. Gureasko, J., W. J. Galush, …, J. Kuriyan. 2008. Membrane-dependent signal integration by the Ras activator Son of sevenless. *Nat. Struct. Mol. Biol.* 15:452–461. https://doi.org/10.1038/nsmb.1418.

41. Huang, W. Y. C., S. Alvarez, …, J. T. Groves. 2021. Relating cellular signaling timescales to single-molecule kinetics: A first-passage time analysis of Ras activation by SOS. *Proc. Natl. Acad. Sci. USA.* 118:e2103598118. https://doi.org/10.1073/pnas.2103598118.

Biophysical Journal, Volume *123*

Supplemental information

Positive feedback in Ras activation by full-length SOS arises from auto-inhibition release mechanism

He Ren, Albert A. Lee, L.J. Nugent Lew, Joseph B. DeGrandchamp, and Jay T. Groves

Supplementary Information for “Positive feedback in Ras activation by full-length SOS arises from autoinhibition release mechanism”

Authors
He Ren[1], Albert A. Lee[2,3], L.J. Nugent Lew[1], Joseph B. DeGrandchamp[1,4], Jay T. Groves[1,5,*]

Affiliations
[1]Department of Chemistry, University of California Berkeley, Berkeley, CA 94720, USA
[2]Department of Molecular and Cell Biology, University of California Berkeley, Berkeley, CA 94720, USA
[3]Current address: Department of Biochemistry, Stanford University, CA 94305, USA
[4]Current address: Institute for Digital Molecular Analytics and Science, Nanyang Technological University, 639798 Singapore.
[5]Division of Molecular Biophysics and Integrated Bioimaging, Lawrence Berkeley National Laboratory, Berkeley, CA 94720, USA.

*Correspondence: jtgroves@lbl.gov

Materials and Methods

Protein purification
LAT-His10 Human LAT cytosolic domain (27-233) was purified with N-terminal 10-Histidine tag (1). The histidine tag was uncleaved for lipid bilayer functionalization.
HCK-His6 Human full length HCK was purified with N-terminal 6-Histindine tag and a TEV site in BL21 transformed with YopH as described in (2). The histidine tag was uncleaved for lipid bilayer functionalization.
SOS_{FL} Human SOS1 was purified though split intein as described in (3).
SOS_{HDPC} The purification of human SOS1 HDPC (1-1049) was described in (4).
SOS_{CAT} The purification of human SOS1 catalytic core (566-1049) was described in (4).
Grb2 Human full length Grb2 was purified with N-terminal 6-Histindine tag and a TEV site as described in (5).
Ras Human H-Ras (1-181, C118S) was purified with N-terminal 6-Histindine tag and a TEV site as described in (3).
RBD sensor RBD-K65E was derived from human Raf1 Ras binding domain with one point mutation (56-131, K65E). It was purified with N-terminal 6-Histine tag, GST, a PreScission site, and a SNAP tag as described in (3).

Protein Labeling
LAT, SOS_{FL}, and SOS_{HDPC} were labeled with Alexa-555 or Alexa-647 C2 Maleimide (Invitrogen). Proteins were diluted to 100 μM or less with 5mM TCEP (Sigma-Aldrich), then react with 1 mM maleimide dye for 2 hr in room temperature. The reaction was then quenched with 5 mM 2-mercaptoethanol (BME, Sigma-Aldrich) for 10 min. Excessive dyes were removed by size exclusion chromatography (SEC) via FPLC (Superdex 75, Cytiva).

RBD sensor was labeled with SNAP-Surface Alexa-488 or Alexa-647 (New England Biolabs). Proteins were mixed with the SNAP-Surface dye at 1:1.5 reaction in PBS and 10 mM DTT at 18 °C overnight. The reaction mixture was buffer exchanged to 20 mM Tris, 200 mM NaCl, 10% glycerol, and 1 mM TCEP before running though SEC to remove excess dye.

Supported lipid bilayer preparation and assay

SUV Small unilamellar vesicles were made using 1,2-dioleoyl-sn-glycero-3-phosphocholine (DOPC), L-α-phosphatidylinositol-4,5-bisphosphate (Brain, Porcine) (ammonium salt) (PI(4,5)P_2), 1,2-dioleoyl-sn-glycero-3-phosphoethanolamine-N-[4-(p-maleimidomethyl)cyclohexane-carboxamide] (sodium salt) (MCC), and 1,2-dioleoyl-sn-glycero-3-[(N-(5-amino-1-carboxypentyl)iminodiacetic acid)succinyl] (nickel salt) (Ni-NTA) from Avanti Polar Lipids. Lipids were mixed in an etched and dried round bottom flask to yield 2 mmol total lipid at 94:2:2:2 (mol%) DOPC:PI(4,5)P_2:MCC:Ni-NTA. After mixing, lipids were dried by rotovap in a 37°C water bath for 2 min then in room temperature for 15 min. Later, the round bottom flask was flushed with N_2 for 15 min. The lipid film was hydrated with 2 mL of milli-Q water for a final concentration of 1 mM total lipids. The cloudy lipid solution was added to a 5 mL polypropylene tube and treated by 3 mm stepped tip sonication at 33% amplitude, 20 s sonication, 50 s rest, 5 cycles (Sonics VCX750) while submerged in ice. The lipid solution was clarified after sonication.

Reaction chamber Precision Schott D 263 M glass coverslips (#1.5H, 75 x 25 mm) was rinsed with milli-Q. Coverslips were then etched with piranha made with 3:1 ratio of concentrated H_2SO_4 (Fisher Scientific) and 30% H_2O_2 (Fisher Scientific) for 5 min and rinse extensively with milli-Q water. To make the experiment chamber, etched coverslips were blow-dried with N_2 and attached to 6-channel flow cells (sticky-Slide VI 0.4, ibidi).

SLB Supported lipid bilayer were made through SUV vesicle fusion. The 1mM lipid solution was diluted to 0.25 mM in PBS (Fisher Scientific). Then, 150 µL of 0.25 mM lipid solution was added to each channel and incubate for 30 min at room temperature. After incubation, the channel was washed with 1mL PBS and blocked with 1mg/mL blocker casein (Thermo Fisher Scientific) in PBS for 10 min. Each channel was washed with 1mL PBS.

Ras bilayer To attach H-Ras on the bilayer, 100 µL of 0.3-0.5 mg/ml H-Ras in reaction buffer (40 mM HEPES (pH 7.4), 100 mM NaCl, 5 mM $MgCl_2$) was added to each channel at room temperature for 2 hr 30 min. Each channel was washed with 1mL PBS and flowed with 100 µL of 5 mM BME in reaction buffer to quench the remaining free maleimide. Each channel was washed with 1 mL PBS again before the Ras bilayer being stored in 100uL of 100 µM guanosine-5'-diphosphate disodium salt (Chem-Impex) in reaction buffer overnight at 4 °C.

LAT bilayer To attach LAT and phosphorylate LAT on the bilayer, the chamber was first taken out of 4 °C, equilibrated to room temperature, and washed with 1mL PBS. Then, 100 µL of 14 nM of LAT and 12.5 nM of HCK were added to each channel for 40 min. After washing the channel with 1 mL PBS, 100 µL of 1 mM of ATP and 100 µM of GDP were added to the channel for LAT phosphorylation and ensure nucleotide loading. Due to the variations in SUV composition, including Ni-NTA lipid composition, LAT densities

vary from day to day, and the variation is essentially unavoidable. Experiments conducted on the same day using the same batch of SUVs usually exhibit minimal variation in LAT density. Therefore, we only compare recruitment traces obtained in the same day.

RasGXP recruitment experiments Due to the intrinsic activity of Ras and supplied GDP in the solution, Ras started as RasGDP on the bilayer. To make RasGTP bilayer, the channel was washed with 1 mL PBS, then 100 µL of 2 nM SOS_{cat}, 1 mM of GTP (Guanosine-5′-Triphosphate Trisodium Salt, Fisher Scientific), and 1 mM of ATP in reaction buffer was flowed into each channel for 30 min. To control for the perturbation brought by SOS_{cat} to the experiment, a RasGDP bilayer was prepared side by side with 100 µL of 2nM SOS_{cat}, 1 mM of GDP, and 1mM of ATP in reaction buffer for 30 min. To wash away SOS_{cat} in the system, 3 mL of PBS with 100 µM GTP or 100 µM GDP was flow through the RasGTP bilayer or RasGDP bilayer respectively. The channel was stored in 1 mM of ATP and 100 µM of GTP or GDP in reaction buffer before any assay. 500 pM of Alexa647-labelled SOS_{CAT}, SOS_{HDPC}, or SOS_{FL} (and 20nM Grb2 for SOS_{FL} exp) was added with 20 nM of Alexa488-labelled RBD, and 1 mM GXP (GTP for RasGTP membrane, GDP for RasGDP membrane) were injected in the flow cell.

Imaging buffer All imaging experiments were ran in imaging buffer with 40 mM HEPES (pH 7.4), 100 mM NaCl, 5 mM MgCl2, 10 mM BME, 100 µM ATP, and 2 mM UV-treated Trolox.

Microscope hardware and imaging acquisition

TIRF imaging experiments were performed on an inverted Nikon Eclipse Ti microscope using a 100X Nikon oil-immersion TIRF objective (1.49 NA), and the 1.5X lens tube were also used in the experiment. 488/561/647 nm diode laser (OBIS laser diode, Coherent) were controlled by a custom built Solemere laser driver to excite fluorophores, and the images were acquired with an iXon Ultra 897 EMCCD camera (Andor Technology). All microscope hardware was controlled using Micro-Manager v4.0. Images were acquired with 15 s time lapse, 500 EM gain, at 100 ms exposure time. High laser intensity (20 mW out of box) was used for single molecule density experiments, and lower laser intensity (2mW) was used for bulk density measurements. For all kinetic traces, middle 250*250 pixels were used to extract data. RBD fluorescence signal were corrected for uneven illumination using shading corrector (Fiji). Single particles were identified with TrackMate (Fiji).

Ras density and LAT density titration

Surface density of Ras and LAT were measured using fluorescence correlation spectroscopy (FCS). The setup was previously described in (6). The FCS imaging experiments were performed on a home-built confocal system on a Nikon TE2000 inverted microscope using a 100x Nikon oil-immersion PlanFluor objective (1.30 NA). A pulsed supercontinuum laser (SuperK Extreme; NKT Photonics) with 100 ps pulse duration at 19.5 MHz repetition rate was used. The signals were recorded by an avalanche photodiode detector (Hamamatsu) and then processed by a hardware correlator (Correlator.com). Then, the autocorrelation function was fitted to a 2D diffusion model to extract surface density and diffusion coefficient. Ras density was measured with Ras nucleotide exchange to EDA-GDP-ATTO-488 (Jena Bioscience), as

described in (6). LAT density was measured with Alexa-555 labeled LAT and corrected for labeling efficiency. A calibration was developed from Ras density measured from FCS and Ras density measured by RBD TIRF signal. Similar LAT density calibration was developed from FCS and TIRF fluorescently labeled LAT signal.

Kinetic simulation

The reaction pathway was listed in Fig. 4A. Since the nucleotide exchange rate (k_{SOS}) of processive SOS_{FL} is nucleotide independent, the fraction of active SOS_{FL} (f(act)) is proportional to the ensemble average catalytic rate:

$$k_{obs} = \frac{\sigma[SOS_{active}] \times k_{SOS} + \sigma[SOS_{inactive}] \times 0}{\sigma[SOS_{active}] + \sigma[SOS_{inactive}]} = \frac{\sigma[SOS_{active}]}{\sigma[SOS_{active}] + \sigma[SOS_{inactive}]} \times k_{SOS} = f(act) \times k_{SOS}$$

Rate equation

The time evolution for the number of each species was solved numerically (ode45 for 3000s) in MATLAB_R2020B.

1. dy(1)/dt=d[SOS_0]/dt=k1*[SOS_solution]-k2*[SOS_0]-k3*[SOS_0]
2. dy(2)/dt=d[SOS_1]/dt= k3*[SOS_0]-k4*[SOS_1]-k5*[RasGTP] *[SOS_1]-k6*[RasGDP] *[SOS_1]
3. dy(3)/dt=d[SOS_1_RasGTP]/dt= k5*[RasGTP] *[SOS_1]-k9*[SOS_1_RasGTP]-k7*[SOS_1_RasGTP]
4. dy(4)/dt=d[SOS_1_RasGDP]/dt= k6*[RasGDP] *[SOS_1]-k10*[SOS_1_RasGDP]- k8*[SOS_1_RasGDP]
5. dy(5)/dt=d[SOS_p_RasGTP]/dt= k7*[SOS_1_RasGTP]-k11*[SOS_p_RasGTP]
6. dy(6)/dt=d[SOS_p_RasGDP]/dt= k8*[SOS_1_RasGDP]-k12*[SOS_p_RasGDP]
7. dy(7)/dt=d[RasGTP]/dt=kcat*([SOS_p_RasGTP]+[SOS_p_RasGDP])*[RasGDP]/[Ras_total]

The following kinetic parameters were used. In next section, the process of how we got the parameters is described.

k1 = k_{on}
k2 = $k_{off\_transient}$
k3 = k_{01}
k4 = $k_{off\_transient}$
k5= $k_{ras\_binding}$ * 6
k6 = $k_{ras\_binding}$
k7 = k_{1p} * **nucleotide dependence**
k8 = k_{1p}
k9 = $k_{off\_transient}$
k10 = $k_{off\_transient}$
k11 = $k_{off\_processive}$
k12 = $k_{off\_processive}$

[SOS_solution]=0.15 nM
[Ras_total]= 2000 μm^{-2}
k_{on} = 0.077 s^{-1} nM^{-1} μm^{-2}
nucleotide dependence = 10
$k_{off\_transient}$ = 0.016 s^{-1}
$k_{off\_processive}$ = 0.01 s^{-1}
k_{01} = 0.01 s^{-1}
$k_{ras\_binding}$ = kon'/ [Ras_total] = 0.3/2000 $s^{-1}molecule^{-1}\mu m^{2}$ (kon' definition see next section)
k_{1p} = 0.01 s^{-1}
k_{cat} = 10 s^{-1}

[SOS_solution] adjustment
The approximated k_{on} from Figure S5 is 0.077*[SOS_solution] $s^{-1}nM^{-1}\mu m^{-2}$, but the binding frequency measurement is specific to the LAT density which varies by each experimental realization. Therefore, we adjusted the [SOS_solution] in our simulations in order to match the SOS membrane recruitment level as the experiment results shown in Figure S6.

$k_{ras\_binding}$ estimation
We approximated $k_{ras\_binding}$ using SOS_{CAT} membrane adsorption kinetics. If assuming SOS_{CAT} membrane binding follows a simple kinetic process as:
SOS + Ras $\xrightarrow{\text{kon}}$ SOS*Ras
SOS*Ras $\xrightarrow{\text{koff}}$ SOS + Ras
By conservation of mass, SOS*Ras + SOS = SOS_total.
$$\frac{d[\mathrm{SOS} * \mathrm{Ras}]}{dt} = \mathrm{kon} \cdot [\mathrm{SOS}] \cdot [\mathrm{Ras}] - \mathrm{koff} \cdot [\mathrm{SOS} * \mathrm{Ras}]$$
Because Ras density is significantly higher than SOS density on the membrane, we can assume:
$$[\mathrm{Ras}] = [\mathrm{Ras_{total}}] - [\mathrm{SOS} * \mathrm{Ras}] \approx [\mathrm{Ras_{total}}]$$
Then the equation can be written as:
$$\mathrm{kon'} \approx \mathrm{kon} \cdot [\mathrm{Ras_{total}}]$$
$$\begin{aligned}\frac{d[\mathrm{SOS} * \mathrm{Ras}]}{dt} &= \mathrm{kon'} \cdot [\mathrm{SOS}] - \mathrm{koff} \cdot [\mathrm{SOS} * \mathrm{Ras}] \\ &= \mathrm{kon'} \cdot (\mathrm{SOS_{total}} - [\mathrm{SOS} * \mathrm{Ras}]) - \mathrm{koff} \cdot [\mathrm{SOS} * \mathrm{Ras}] \\ &= \mathrm{kon'} \cdot \mathrm{SOS_{total}} - \mathrm{kon'} \cdot [\mathrm{SOS} * \mathrm{Ras}] - \mathrm{koff} \cdot [\mathrm{SOS} * \mathrm{Ras}] \\ &= constant - (\mathrm{kon'} + \mathrm{koff}) \cdot [\mathrm{SOS} * \mathrm{Ras}]\end{aligned}$$
$$[\mathrm{SOS} * \mathrm{Ras}] = constant * (1 - e^{-(\mathrm{kon'}+\mathrm{koff})t})$$
For both RasGTP and RasGDP, SOScat reaches equilibrium before 15s. We can fit the above equation to t=15, normalized SOScat recruitment = $\frac{[\mathrm{SOS}*\mathrm{Ras}]}{constant}$ =0.9999. Then $\mathrm{kon'} + \mathrm{koff}$ is around 0.6 s^{-1}. Since SOScat binding affinity (K_D) toward both RasGTP and RasGDP are in the micromolar range (7,8) in solution, we expect kon to be bigger than koff on membrane. Therefore, we varied kon' from 0.3, 3, to 30 and found that

because Ras binding is not a rate limiting step, there is very little difference between 0.3, 3, and 30, shown in Figure S7.

Additionally, to differentiate the RasGTP and RasGDP binding pathway, we varied the rate according to the difference in binding affinity of RasGTP (K_D ~3.6 μM) and RasGDP (K_D ~24.5 μM) to SOScat allosteric binding pocket (7). By assuming the difference in binding affinities primarily presents in the on rate, we concluded k5=6*k6.

k_{01} and k_{1p} estimation

Earlier single molecular measurements (3) have shown that the timescale for SOS getting into the active state is tens of seconds. k_{01} and k_{1p} determine the rate of SOS activation and the timescale for the bulk nucleotide reaction to be done. For simplicity, we define $k_{01}=k_{1p}$. Then, we varied k_{01} and k_{1p} from 0.05, 0.02, 0.01, and 0.005, and found 0.01 best reproduced experimental results, shown in Figure S8.

k_{cat} estimation

We estimated kcat to be 10 s^{-1} based on Figure 3D.

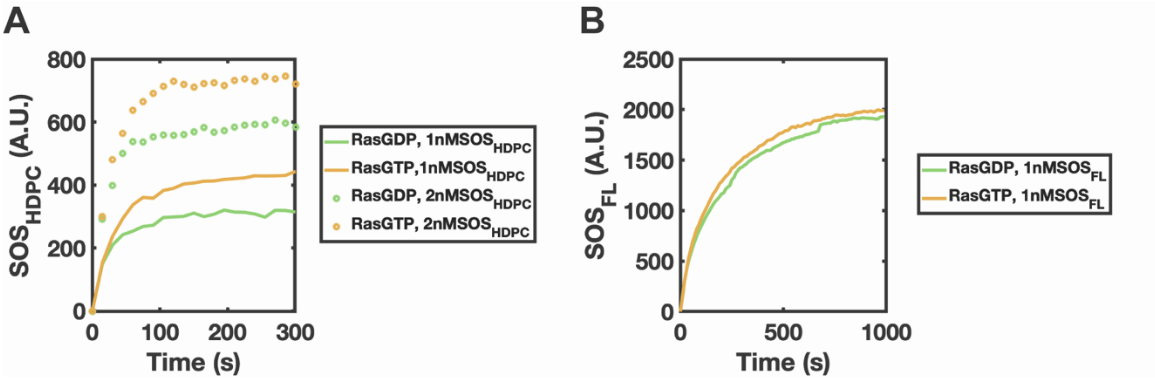

Figure S1. Additional SOS recruitment traces. Consistent with Figure 2, (A) SOS_{HDPC} membrane recruitment is Ras nucleotide dependent (N=1). (B) LAT:Grb2 mediated SOS_{FL} membrane recruitment is not Ras nucleotide dependent (N=1). Due to the high amount of SOS membrane recruitment, SOS density cannot be resolved by single particle counting in these experiments. Bulk fluorescence intensity, which is proportional to particle density, is shown in the y axis.

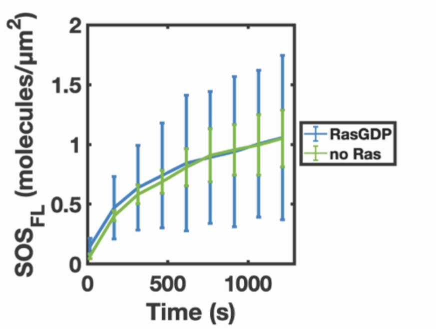

Figure S2. The initial SOS_{FL} recruitment kinetics is independent of Ras density. We did not observe measurable effects in the initial SOS recruitment kinetics caused by the presence of Ras (N=2). Since Ras binding is involved in SOS ultimately reaching its catalytically active state, there is an intrinsic difference in the overall state of membrane-recruited SOS with or without membrane Ras. This naturally suggests that, in equilibrium, the overall amount of membrane recruited SOS will depend on Ras. The SOS_{FL} membrane recruitment experiments, and likely the physiological system as well, however, do not examine equilibrium conditions. Instead, the results are driven by kinetics of the initial recruitment process, and these are dominated by Grb2.

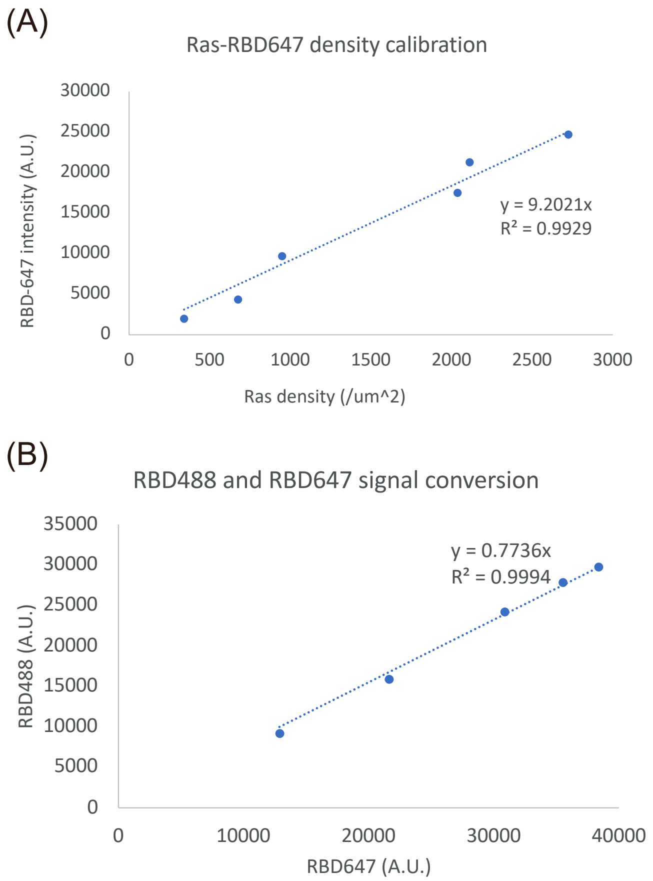

Figure S3. Ras density and RBD sensor calibration. (A) Ras density was measured by FCS using Ras with fluorescently labeled nucleotides. The same Ras bilayer was measured with RBD-Ax647, and the intensity was obtained from TIRFM. (B) RBD-Ax647 and RBD-Ax488 were added to the Ras bilayer simultaneously, and both signal intensities were measured with TIRFM.

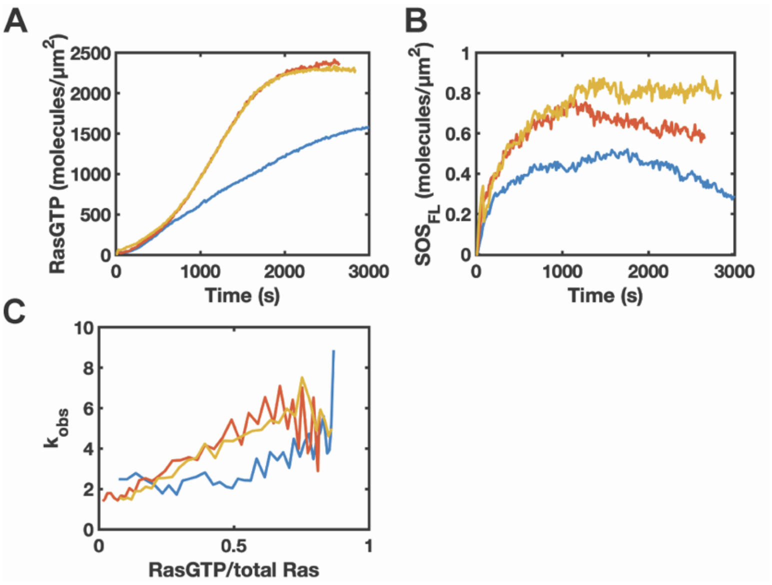

Figure S4. Ras activation and SOS membrane recruitment heterogeneity. Three representative traces for Ras activation (A), SOS membrane recruitment (B), and k_{obs} calculation.

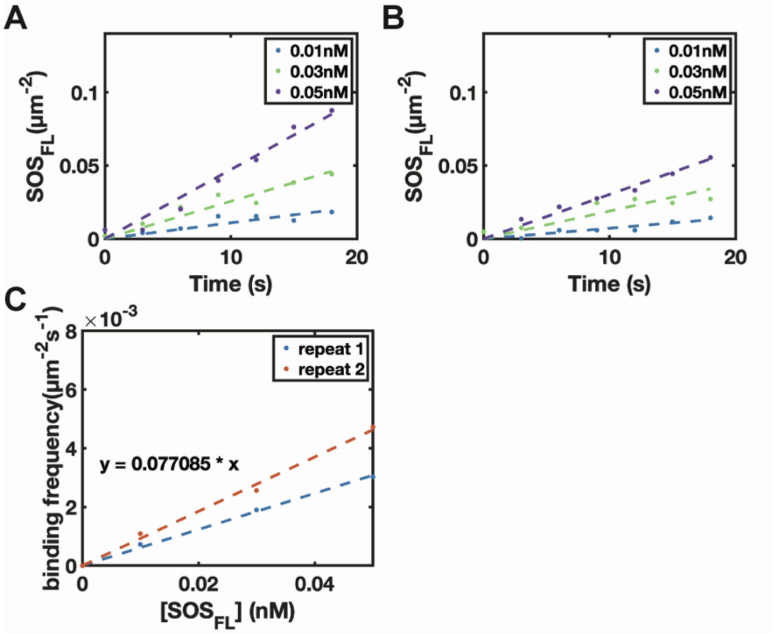

Figure S5. SOS_{FL} membrane recruitment rate estimation on a Ras bilayer with LAT and Grb2. Two repeats (A and B) for SOS_{FL} membrane recruitment at early time points. (C) The concentration-dependent binding frequency of SOS_{FL} to a Ras bilayer.

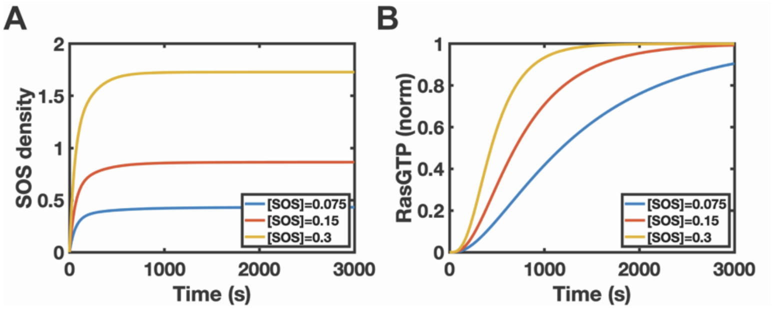

Figure S6. SOS concentration adjustment in simulation. [SOS_solution] = 0.15 works the best.

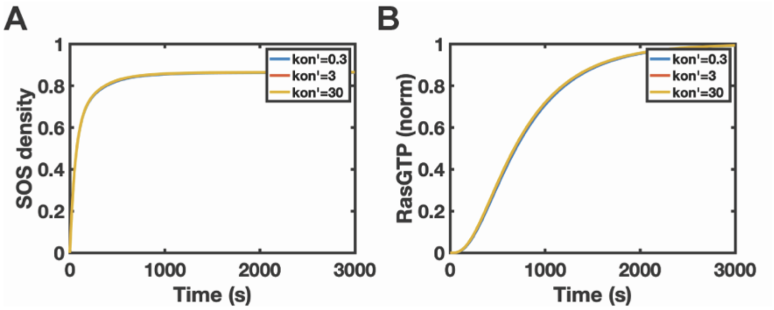

Figure S7. $k_{ras\_binding}$ estimation. Variation in kon' (or $k_{ras\_binding}$=kon'/[Ras_total]) does not have significant effects on SOS recruitment and Ras activation.

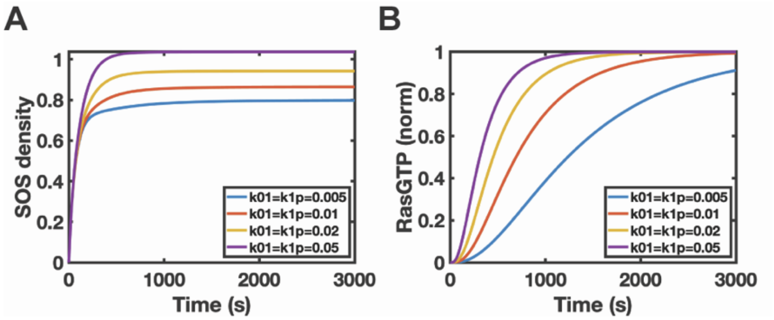

Figure S8. k_{01} and k_{1p} estimation. Various rates were tested, and k_{01}=k_{1p}=0.01 plot aligns with experimental measurement (Figure 3) the best.

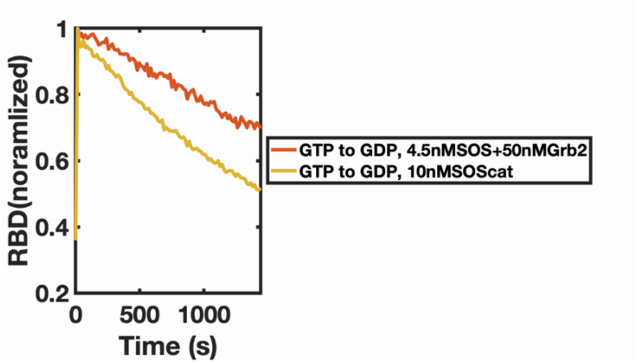

Figure S9. RasGTP to RasGDP nucleotide exchange reaction. First of all, it is not the natural reaction for SOS and the catalytic rate of SOS does exhibit nucleotide effects when swapping in GDP instead of GTP (9). Secondly, the SOS_FL autoinhibition release kinetics and dissociation rate once activated are quite slow, suggesting the reaction is not likely to exhibit significant non-equilibrium hysteresis effects even if it could be run symmetrically in reverse.

Reference

1. Cho Nathan, H., C. Cheveralls Keith, A.-D. Brunner, K. Kim, C. Michaelis André, P. Raghavan, H. Kobayashi, L. Savy, Y. Li Jason, H. Canaj, Y. S. Kim James, M. Stewart Edna, C. Gnann, F. McCarthy, P. Cabrera Joana, M. Brunetti Rachel, B. Chhun Bryant, G. Dingle, Y. Hein Marco, B. Huang, B. Mehta Shalin, S. Weissman Jonathan, R. Gómez-Sjöberg, N. Itzhak Daniel, A. Royer Loïc, M. Mann, and D. Leonetti Manuel. OpenCell: Endogenous tagging for the cartography of human cellular organization. *Science*. 375(6585):eabi6983, doi: 10.1126/science.abi6983
2. Seeliger, M. A., M. Young, M. N. Henderson, P. Pellicena, D. S. King, A. M. Falick, and J. Kuriyan. 2005. High yield bacterial expression of active c-Abl and c-Src tyrosine kinases. *Protein Science*. 14(12):3135-3139, doi: https://doi.org/10.1110/ps.051750905
3. Huang, W. Y. C., S. Alvarez, Y. Kondo, Y. K. Lee, J. K. Chung, H. Y. M. Lam, K. H. Biswas, J. Kuriyan, and J. T. Groves. 2019. A molecular assembly phase transition and kinetic proofreading modulate Ras activation by SOS. *Science*. 363(6431):1098-1103, doi: 10.1126/science.aau5721
4. Gureasko, J., W. J. Galush, S. Boykevisch, H. Sondermann, D. Bar-Sagi, J. T. Groves, and J. Kuriyan. 2008. Membrane-dependent signal integration by the Ras activator Son of sevenless. *Nature Structural & Molecular Biology*. 15(5):452-461, doi: 10.1038/nsmb.1418
5. Lin, C.-W., L. M. Nocka, B. L. Stinger, J. B. DeGrandchamp, L. J. N. Lew, S. Alvarez, H. T. Phan, Y. Kondo, J. Kuriyan, and J. T. Groves. 2022. A two-component protein condensate of the EGFR cytoplasmic tail and Grb2 regulates Ras activation by SOS at the membrane. *Proceedings of the National Academy of Sciences*. 119(19):e2122531119, doi: 10.1073/pnas.2122531119
6. Chung, J. K., Y. K. Lee, H. Y. M. Lam, and J. T. Groves. 2016. Covalent Ras Dimerization on Membrane Surfaces through Photosensitized Oxidation. *Journal of the American Chemical Society*. 138(6):1800-1803, doi: 10.1021/jacs.5b12648
7. Sondermann, H., S. M. Soisson, S. Boykevisch, S. S. Yang, D. Bar-Sagi, and J. Kuriyan. 2004. Structural analysis of autoinhibition in the Ras activator Son of sevenless. *Cell*. 119(3):393-405, doi: 10.1016/j.cell.2004.10.005
8. Vo, U., N. Vajpai, L. Flavell, R. Bobby, A. L. Breeze, K. J. Embrey, and A. P. Golovanov. 2016. Monitoring Ras Interactions with the Nucleotide Exchange Factor Son of Sevenless (Sos) Using Site-specific NMR Reporter Signals and Intrinsic Fluorescence*. *Journal of Biological Chemistry*. 291(4):1703-1718, doi: https://doi.org/10.1074/jbc.M115.691238
9. Iversen, L., H.-L. Tu, W.-C. Lin, M. Christensen Sune, M. Abel Steven, J. Iwig, H.-J. Wu, J. Gureasko, C. Rhodes, S. Petit Rebecca, D. Hansen Scott, P. Thill, C.-H. Yu, D. Stamou, K. Chakraborty Arup, J. Kuriyan, and T. Groves Jay. 2014. Ras activation by SOS: Allosteric regulation by altered fluctuation dynamics. *Science*. 345(6192):50-54, doi: 10.1126/science.1250373
